# Cylindracin, a Cys‐rich protein expressed in the fruiting body of *Cyclocybe cylindracea*, inhibits growth of filamentous fungi but not yeasts or bacteria

**DOI:** 10.1002/2211-5463.13910

**Published:** 2024-10-08

**Authors:** Yamato Kuratani, Chika Abematsu, Keisuke Ekino, Takuji Oka, Masashi Shin, Makoto Iwata, Hiroto Ohta, Shoji Ando

**Affiliations:** ^1^ Faculty of Biotechnology and Life Science Sojo University Kumamoto Japan; ^2^ IMB Co., Ltd. Asakura Japan

**Keywords:** antifungal protein, Basidiomycetes, *Cyclocybe cylindracea*, cylindracin, host defense, Pri3

## Abstract

Mushrooms are the fruiting bodies of fungi and are important reproductive structures that produce and disseminate spores. The *Pri3* gene was originally reported to be specifically expressed in the primordia (a precursor to the mature fruiting body) of the edible mushroom *Cyclocybe aegerita*. Here, we cloned a *Pri3*‐related cDNA from *Cyclocybe cylindracea*, another species in the same genus, and showed that the gene is specifically expressed at the pileus surface of the immature fruiting body but not in the primordia. Immunohistochemistry showed that the translated protein is secreted into a polysaccharide layer of the pileus surface. The recombinant *C*‐terminal Cys‐rich domain of the protein showed antifungal activity against three filamentous fungi and inhibited hyphal growth and conidiogenesis. These results suggest that the PRI3‐related protein of *C. cylindracea*, named cylindracin, plays an important role in the defense against pathogens.

## Graphical abstract

This study demonstrates that cylindracin, a cationic cysteine‐rich protein, is specifically expressed in the immature fruiting body of the edible mushroom *Cyclocybe cylindracea*. The recombinant cylindracin showed antifungal activity against filamentous fungi and inhibited hyphal growth and conidiogenesis, through damaging the fungal plasma membranes and cell walls. The data suggest that cylindracin is important in host defense against pathogenic invaders.

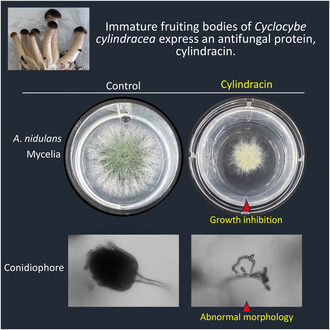

**Abbreviations**
CFDA: carboxyfluorescein diacetate
CFW: calcofluor‐white
DIG: digoxigenin
PBS: phosphate‐buffered saline
PDA: potato dextrose agar
PDB: potato dextrose broth
PI: propidium iodide
SDS/PAGE: sodium dodecyl sulfate‐polyacrylamide gel electrophoresis
SSC: saline sodium citrate
YPD: yeast extract peptone dextrose
YPG: yeast extract peptone glycerol
YPM: yeast extract peptone methanol

Mushrooms, the fruiting bodies of Basidiomycetes and some Ascomycetes, are widely consumed for their taste and aroma, and also for their nutritional and medicinal value [1, 2, 3, 4]. The medicinal properties include antibacterial [5, 6, 7, 8, 9, 10, 11], antifungal [5], antiviral [5, 9, 12, 13], antioxidant [5, 9], and antineoplastic activities [9, 12, 13]. These medicinal properties of the mushroom fruiting bodies have been assigned to polysaccharides [9, 12, 13], secondary metabolites [6, 12, 13], and peptides and proteins [5, 6, 7, 8, 10, 11]. Exploration of as‐yet‐unknown bioactive compounds in mushroom fruiting bodies and elucidation of the mechanisms underlying their biological functions are ongoing worldwide to develop new functional medicines and agrochemicals.

Fruiting bodies are important reproductive structures that produce and disseminate spores [1, 4, 14]. Molecular mechanisms underlying fruiting body formation of mushrooms have been investigated by genetic and proteomic approaches using representative model mushrooms, including *Coprinopsis cinerea* [4], *Schizophyllum commune* [15], *Lentinula edodes* [16, 17], and *Agrocybe aegerita* (synonym: *Cyclocybe aegerita*) [18, 19, 20]. To initiate fruiting body formation, spores germinate hyphae, which are chains of filamentous cells that branch and anastomose to each other, to form a root‐like structure called the mycelium. Upon receiving the appropriate environmental signals such as drop in temperature or exhaustion of nutrition, mycelia differentiate into many types of different cells and associate with each other to form hyphal knots (~ 0.2 mm in size) that further develop into bipolar primordia (2 to several mm in height) composed of distinctive cellular tissues to form the stipe, pileus, and gills in the mature fruiting body [1, 4, 14]. A cluster of parallel‐oriented hyphae close to the primordial base forms the stipe of the fruiting body, while the apical tissue of the primordia develops into the pileus. During stipe elongation and pileus maturation, basidiospore formation proceeds within the basidia located on the surface of the gills that form on the underside of the pileus [1, 4]. Fully opened pileus and distinctly formed gills with basidiospores indicate maturation of the fruiting body.

The *Aa1*‐*Pri3* gene has been reported to be expressed specifically in the primordia of the edible mushroom *A. aegerita* [21]; it was named after the third gene identified by the authors to be specifically expressed in primordia, where the first two letters represent the abbreviated species name and the number is the strain number (i.e., *Aa1*). Homologous genes have been identified in seven other strains of *A*. *aegerita* as well as in a strain of *A*. *chaxingu*, another species in the same genus, by PCR of genomic DNAs extracted from mycelia [21]. The deduced amino acid sequences were 95 amino acids long, including a putative *N*‐terminal signal peptide of 20 amino acids, and a *C*‐terminal sequence of 75 amino acids, including eight Cys residues at conserved positions. However, physiological functions of PRI3 proteins remain unknown [21]. Later, an antifungal protein with a molecular weight of 9 kDa and named “agrocybin” was isolated from the fruiting body of *A. cylindracea*, another species in the genus *Agrocybe* [22]; the *N*‐terminal 15 residues of this protein were homologous to the sequence from Ala‐37 to Phe‐51 of the protein deduced from the *Ac‐Pri3* gene of *A*. *chaxingu* [21]. However, the relationship between agrocybin and the *Pri3* gene‐encoded proteins remains unclear. Recently, the species *A. aegerita*, *A. chaxingu*, and *A. cylindracea* were moved from the genus *Agrocybe* into the resurrected genus *Cyclocybe* on the basis of a phylogenic study based on comparisons of rDNA sequence data; therefore, they are currently referred to as *C. aegerita*, *C. chaxingu*, and *C. cylindracea*, respectively [20, 23, 24].

In this study, we cloned a *Pri3*‐related cDNA from *C. cylindracea* and identified the expression site of the gene in the fruiting body. We characterized a biological function of the gene product using recombinant protein. The results indicated that the *Pri3*‐related gene is specifically expressed in the pileus of the immature fruiting body, but not in the primordia of *C. cylindracea*. Immunohistochemical analysis suggested that the gene product is secreted into a polysaccharide‐containing layer of the pileus surface. Recombinant *C*‐terminal Cys‐rich domain of the PRI3‐related protein inhibited hyphal growth and conidiogenesis of filamentous fungi, but it did not inhibit proliferation of bacteria or yeasts. The results also suggest that agrocybin [22] is a post‐translationally processed product of a *Pri3*‐related gene in *C. cylindracea*. We propose here to name the PRI3‐related protein from *C. cylindracea* “cylindracin.”

## Materials and methods

### Cultivation of mycelia and fruiting bodies

Mycelia and fruiting bodies of *C. cylindracea* IMBYNG1 strain were obtained from IMB Co., Ltd. (Asakura City, Japan). Vegetatively growing mycelia were cultured in Ohta's medium [1% (w/v) glucose, 0.1% (w/v) citric acid, 0.1% (w/v) ammonium tartrate, 0.1% (w/v) KH_2_PO_4_, 0.1% (w/v) MgSO_4_·7H_2_O, 0.005% (w/v) CaCl_2_·2H_2_O, 0.7% (w/v) HEPES‐KOH, pH 5, 1% (v/v) mineral solution, 1% (v/v) vitamin solution] [25] at 25 °C for 2 months and then harvested by filtration over gauze, washed with an isotonic sucrose solution, frozen immediately in liquid nitrogen, and stored at −80 °C. For fruiting body formation, the mycelia of *C. cylindracea* were cultivated on packs of sawdust compost (40% sawdust, 15% rice bran, 45% tap water) at 22 °C. Once the mycelia had fully covered the compost, they were scratched with a dispensing spoon and then subjected to cultivation at 18 °C for several days to allow primordium initiation. Primordia with height of about 5 mm, immature fruiting bodies with height of 2–4 cm, and mature fruiting bodies with height of about 8 cm were harvested separately, frozen in liquid nitrogen, and stored at −80 °C.

### Other microorganisms

*Aspergillus nidulans* FGSC A4 was provided by the Fungal Genetics Stock Center (University of Kansas Medical Center, Kansas City, KS, USA). *Alternaria porri* MAFF237561 was purchased from the Research Center of Genetic Resources (National Agriculture and Food Research Organization, Tsukuba, Japan). *Fusarium oxysporum* f. sp. *cepae* 205 was provided by Yamaguchi University, Yamaguchi City, Japan [26]. The yeasts *Schizosaccharomyces japonicus* NRRL Y‐1026E and *Saccharomyces cerevisiae* sigma 1278b were provided by Sojo University, Kumamoto City, Japan. *Escherichia coli* DH5α and *Micrococcus luteus* were purchased from Nippon Gene (Tokyo, Japan) and MP Biomedicals (Santa Ana, CA, USA), respectively. *Pichia pastoris* strains X‐33 and SMD1168H were purchased from Thermo Fischer Scientific (Waltham, MA, USA).

### Total RNA isolation and cDNA cloning

Frozen mycelia and fruiting bodies that were cut into small pieces were smashed into powder in liquid nitrogen and used for RNA extraction. Total RNA was extracted from the samples using a PureLink RNA Mini Kit (Thermo Fischer Scientific) according to the manufacturer's instructions. The concentration of the RNA samples was determined spectrophotometrically at 260 nm. First‐strand cDNA synthesis was performed with total RNA using the EasyScript Plus cDNA Synthesis Kit (Applied Biological Materials, Richmond, BC, Canada) and an adaptor‐dT primer (5′‐GGCCACGCGTCGACTAGTACTTTTTTTTTTTTTTTTT‐3′), according to the manufacturer's instructions. *Pri3*‐related cDNA was amplified by nested PCR using the first‐strand cDNA, the adaptor‐dT primer and two kinds of *Pri3*‐specific forward primers, Fw‐1 (5′‐TCCCTTCAAGAAAGTGCATC‐3′) and Fw‐2 (5′‐GACCGTCAACTGGATATCCC‐3′), respectively based on the genome sequence of *C. cylindracea* (https://www.ncbi.nlm.nih.gov/datasets/genome/GCA_013376435.1/) [27]. Information about the exon/intron boundaries in the 5′‐terminal untranslated sequence was obtained from the genome sequence of the *Ac‐Pri3* gene (AF369627) from *C. chaxingu* [21]. After blunting kination reaction, the cDNA, hereinafter referred to as *Cc‐Pri3*, was cloned into plasmid pUC118 HincII/BAP (Takara Bio, Kusatsu, Japan), propagated using *E. coli* DH5α, and characterized by DNA sequencing.

### Northern blotting

Digoxigenin (DIG)‐labeled antisense and sense RNA probes (289 nt) specific for *Pri3* mRNA were prepared using T7 RNA polymerase, the DIG RNA Labeling Mix (Sigma‐Aldrich, St. Louis, MO, USA), and template cDNA (310 nt) containing the T7 RNA promoter sequence (18 nt) followed by the antisense or sense sequence of the *Cc*‐*Pri3* cDNA, corresponding to nucleotide residues 154–436 in Fig. S1. The template cDNA for the antisense RNA probe was prepared by PCR using pUC118‐*Cc*‐*Pri3* as the template, a forward primer [Pri3 (154–177)‐antisense probe: 5′‐GAGGCGCTAGAAGGCCGCGCTAAC‐3′] and a reverse primer (T7‐Y450‐antisense probe: 5′‐GAGATAATACGACTCACTATAGGGAGAGAAGTTTCGAAACGCACACCCCTT‐3′) containing the T7 promoter sequence (underlined). The template cDNA for the sense RNA probe was prepared by PCR using pUC118‐*Cc*‐*Pri3* as the template, a forward primer [T7‐Pri3 (154–177)‐sense probe: 5′‐GAGATAATACGACTCACTATAGGGAGAGAGGCGCTAGAAGGCCGCGCTAAC‐3′] containing the T7 promoter sequence (underlined), and a reverse primer (Y450‐R416: 5′‐GAAGTTTCGAAACGCACACCCCTTCGAGCA‐3′).

Total RNAs (5–20 μg) extracted from mycelia, primordia, fruiting body stem, or fruiting body pileus were electrophoresed on a 1.2% agarose gel containing 0.66 m formaldehyde and then transferred to a nylon membrane, Hybond‐N^+^ (GE Healthcare, Chicago, IL, USA). DynaMarker RNA High (BioDynamics Laboratory Inc., Tokyo, Japan) was used to indicate molecular weights. After irradiation with ultraviolet light, the membrane was prehybridized in DIG Easy Hyb solution (Sigma‐Aldrich) at 68 °C for 3 h and then hybridized with the DIG‐labeled RNA sense or antisense probe (100 ng·mL^−1^) in DIG Easy Hyb solution at 68 °C overnight. After washing, the membrane was incubated with alkaline phosphatase‐conjugated anti‐DIG antibody Fab fragment (Sigma‐Aldrich), diluted 1 : 20 000, at 25 °C for 30 min. After washing, immunoreactive bands were visualized using CDP‐star (Sigma‐Aldrich), an alkaline phosphatase substrate.

### *In situ* hybridization

Fruiting body stems and pilei were cut to a suitable size (25 × 18 × 2 mm), fixed in 0.1 m potassium phosphate buffer (pH 7.4) containing 4% paraformaldehyde and 0.25% glutaraldehyde, washed with 0.1 m potassium phosphate (pH 7.4), and stored at 4 °C until the next step. After dehydration with an ethanol series, samples were incubated in xylene, embedded in paraffin, sectionized at 5‐μm thickness, and mounted on silane‐coated slides. After removing paraffin with xylene and then rehydration with an ethanol series, samples were treated with 0.3% Triton X‐100 in phosphate‐buffered saline (PBS) at room temperature for 30 min and then with proteinase K (10 μg·mL^−1^) in 0.1 m Tris–HCl (pH 7.5) containing 50 mm ethylenediaminetetraacetic acid (EDTA) at 37 °C for 30 min. Acetylation was performed with 0.25% (v/v) acetic anhydride in 1.33% (v/v) triethanolamine‐HCl (pH 8.0) at room temperature for 15 min. Prehybridization was performed in 5‐times concentrated saline sodium citrate buffer (5× SSC; 0.75 m NaCl, 75 mm trisodium citrate, pH 7.0) containing 50% (v/v) formamide, 5× Denhardt's solution [28], baker's yeast RNA (250 μg·mL^−1^; Sigma‐Aldrich), and salmon sperm DNA (500 μg·mL^−1^; Fujifilm Wako Chemicals, Osaka, Japan), at 37 °C for 2 h. Hybridization was performed in the buffer used for prehybridization supplemented with 5% (w/v) dextran sulfate and DIG‐labeled RNA probe (0.2–1.0 μg·mL^−1^), at 65 °C, overnight. After washing with 5× SSC and 0.2× SSC, blocking was performed in 85 mm Tris–HCl (pH 7.5) containing 0.13 m NaCl and 1.5% blocking reagent (Sigma‐Aldrich) at room temperature for 1 h. To detect the hybridized DIG‐labeled probe, samples were incubated in the blocking solution containing an alkaline phosphatase‐conjugated anti‐DIG antibody Fab fragment (Sigma‐Aldrich), diluted 1 : 250, at room temperature, for 1 h. Signals were detected by incubation with nitro blue tetrazolium (NBT, 200–450 μg·mL^−1^; Sigma‐Aldrich) and 5‐bromo‐4‐chrolo‐3‐indoyl phosphate (BCIP, 175 μg·mL^−1^; Sigma‐Aldrich) in buffer containing 100 mm Tris–HCl (pH 9.5), 50 mm MgCl_2_, and 100 mm NaCl. Color development was stopped by exchanging the color development solution with 10 mm Tris–HCl buffer (pH 7.5) containing 1 mm EDTA. Specimens were examined using an Olympus BX53 microscope (Tokyo, Japan).

### Preparation of antibodies

**Fig. 1:**
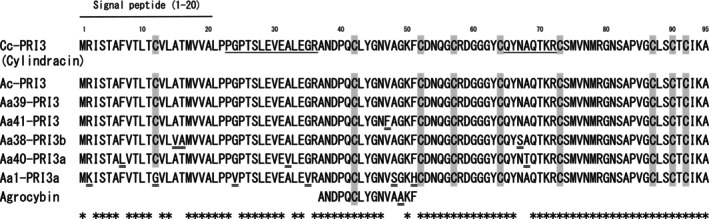
Amino acid sequence alignment of Cc‐PRI3 with other PRI3‐related proteins. Cc‐PRI3 (cylindracin: alternatively proposed name in this study) has the same amino acid sequence as the sequence deduced from the *Ac‐Pri3* gene (AF369627) in *Cyclocybe chaxingu* (syn. *Agrocybe chaxingu*) [20, 21]. Amino acid substitutions among the PRI3 proteins are indicated using a double underline. The *N*‐terminal 20 residues of the PRI3 proteins are predicted to be signal peptides [37]. Conserved cysteine residues are highlighted by a gray background. The sequences of synthetic peptides used as immunogens for production of antisera are indicated by underlines. Database accession numbers: *Aa39‐Pri3*, AF369623, AF369630; *Aa41‐Pri3*, AF369626, AF369632; *Aa38‐Pri3b*, AF369622, AF369629; *Aa40‐Pri3a*, AF369624, AF369631; *Aa1‐Pri3a*, AF369616, AF369634–369640. The *N*‐terminal 15 residues of agrocybin have been reported [22].

Rabbit antisera against synthetic peptides [CPGPTSLEVEALEGR or C(Ahx)QYNAQTKR, Ahx: aminohexanoic acid] representing residues 23–36 or 65–72 of the Cc‐PRI3 protein (Fig. 1) and containing a cysteine residue at the *N*‐terminus were raised by Eurofins Genomics (Tokyo, Japan). For antibody affinity purification, the peptide was coupled to SulfoLink Coupling Resin (Thermo Fisher Scientific). The antiserum was applied to the matrix, which was then washed with PBS. The specific antibody was eluted with 100 mm glycine buffer (pH 2.5), neutralized by 1 m Tris–HCl (pH 7.5), and then dialyzed against PBS.

### Western blotting

Pileus of fruiting body of about 4 cm height was frozen in liquid nitrogen, ground in a mortar using a pestle, suspended in 10 mm Tris–HCl buffer (pH 8.8) containing 0.03% CHAPS, 1 mm phenylmethylsulfonyl fluoride, 1 μg·mL^−1^ leupeptin, and protease inhibitor cocktail, and then stirred on ice for 1 h. After filtration using gauze, the filtrate was centrifuged at 15 000 ***g*** at 4 °C for 1 h. The supernatant was treated with 40% ammonium sulfate, then centrifuged at 15 000 ***g*** at 4 °C for 30 min. The supernatant was treated with 80% ammonium sulfate, then centrifuged at 15 000 ***g*** at 4 °C for 30 min. Proteins in the precipitates from the salting‐out steps were dissolved in 20 mm HEPES‐NaOH (pH 7). An aliquot of the sample was heat‐treated in sample buffer containing 5 mm dithiothreitol for sodium dodecyl sulfate‐polyacrylamide gel electrophoresis (SDS/PAGE), loaded on 18% polyacrylamide gels, and resolved by SDS/PAGE. The proteins were transferred onto polyvinylidene difluoride membranes (Thermo Fisher Scientific) and detected using the purified rabbit antibodies raised against synthetic peptides, with alkaline phosphatase‐conjugated goat anti‐rabbit IgG (Abcam, Cambridge, UK) as the secondary antibody and CDP‐star as an alkaline phosphatase substrate.

### Immunohistochemistry

Thin sections of fruiting body mounted on silane‐coated slides were permeabilized with 0.2% Triton X‐100 in PBS for 2 min. After washing in PBS, the specimens were incubated in Block Ace blocking solution (Sumitomo Dainippon Pharma, Osaka, Japan) for 30 min. The specimens were then incubated with the purified antibody raised against the synthetic peptide representing residues 23–36 of the Cc‐PRI3 protein (diluted 1 : 200 in tenfold‐diluted blocking solution) at 4 °C overnight, then washed three times with PBS for 5 min each. The specimens were incubated with the secondary antibody used for western blotting at room temperature for 2 h. After washing in PBS, immunohistochemical signals were detected by incubation with NBT and BCIP, and color development was stopped by washing with 10 mm Tris–HCl buffer (pH 7.5) containing 1 mm EDTA. Cell nuclei and cytoplasm were stained with Nuclear Fast Red solution (ScyTek Laboratories, Logan, UT, USA). For characterization of the surface components of the pileus, Alcian blue solution (Fujifilm Wako Chemicals) was used to detect acidic polysaccharides [29]. Specimens were examined using an Olympus BX53 microscope.

### Recombinant PRI3 protein preparation

The DNA encoding residues 37–95 of the Cc‐PRI3 protein was codon‐optimized based on codon usage in *Pichia pastoris* and was chemically synthesized (Eurofins Genomics); restriction sites for *Eco*RI and *Xba*I were added to the 5′‐ and 3′‐terminal ends, respectively. The resulting recombinant protein, referred to as 6 × His‐Cc‐PRI3(37–95) (total amino acids 79, calculated molecular weight 8641.56), has the extra *N*‐terminal sequence EFHHHHHHGEVELEVLFQGP, including a 6 × His‐tag sequence (underline) followed by the HRV‐3C protease recognition sequence (double underline) [30]. The synthetic insert DNA and a *Pichia* expression vector, pPICZαA (Thermo Fisher Scientific), were ligated via the *Eco*RI and *Xba*I restriction sites. The resultant plasmid, pPICZαA‐6 × His‐Cc‐PRI3(37–95), was propagated using *E. coli* DH5α, and confirmed by restriction analysis and DNA sequencing.

The plasmid pPICZαA‐6 × His‐Cc‐PRI3(37–95) was linearized using *Sac*I and transformed by electroporation into *P. pastoris* SMD1168H cells (lacking the *pep4* gene that encodes proteinase A) [31]. The transformants were grown on YPDS plates [1% (w/v) yeast extract, 2% (w/v) peptone, 2% (w/v) glucose, 1 m sorbitol, 2% (w/v) agar] containing 100 μg·mL^−1^ Zeocin (Thermo Fisher Scientific). The clones containing the *Cc‐Pri3* gene were identified by colony PCR using primer set AOX1‐Fw (5′‐GACTGGTTCCAATTGACAAGC‐3′) and AOX1‐Rv (5′‐GCAAATGGCATTCTGACATCC‐3′).

The *Pichia* clone expressing recombinant protein was pre‐cultivated in 5 mL of yeast extract peptone dextrose (YPD) medium [1% (w/v) yeast extract, 2% (w/v) peptone, 2% (w/v) glucose] containing 100 μg·mL^−1^ Zeocin until the optical density at 660 nm exceeded 1.7. All the culture broth was used to inoculate 3 L of YPG complex medium [100 mm potassium phosphate (pH 6.0), 1% (w/v) yeast extract, 2% (w/v) peptone, 1% (v/v) glycerol, 1.34% (w/v) yeast nitrogen base without amino acids, 4 × 10^−5^% (w/v) biotin] in a fermenter (Mitsuwa Frontech, Osaka, Japan), and then cultivation was performed at 30 °C for 72 h at an agitation speed of 120 rpm and an aeration rate of 3 L·min^−1^. After centrifugation at 3000 ***g*** for 10 min at 4 °C, the cell pellets were resuspended in 3 L of YPM complex medium [100 mm potassium phosphate (pH 6.0), 1% (w/v) yeast extract, 2% (w/v) peptone, 0.5% (v/v) methanol, 1.34% (w/v) yeast nitrogen base without amino acids, 4 × 10^−5^% (w/v) biotin], and cultivated at 30 °C for 48 h at agitation speed of 120 rpm and aeration rate of 3 L·min^−1^. Twenty‐four hours after starting cultivation, methanol was added once more into the broth to restore the methanol concentration to 0.5%. After centrifugation at 3000 ***g*** for 20 min at 4 °C, the supernatant was passed through a filter of 0.45‐μm pore‐size (Merck Millipore, Burlington, MA, USA). Recombinant protein in the supernatant was precipitated by addition of ammonium sulfate at a final concentration of 90% saturation, collected by centrifugation at 10 000 ***g*** for 40 min at 4 °C, redissolved in buffer containing 20 mm HEPES‐NaOH (pH 7.0), 150 mm NaCl, and 5 mm imidazole, and then loaded onto a HisPur Cobalt Superflow Agarose column (1.6 × 4.5 cm; Thermo Fisher Scientific) that was pre‐equilibrated with the same buffer. After washing the column with the pre‐equilibration buffer, recombinant protein was eluted from the column with 20 mm HEPES‐NaOH (pH 7.0), 150 mm NaCl, and 500 mm imidazole. The recombinant protein was concentrated by salting out using 80% ammonium sulfate and then desalted using a Zeba Spin column with a molecular weight cut‐off of 7 kDa (Thermo Fisher Scientific) that was prefilled with 20 mm HEPES‐NaOH (pH 7.0). The obtained recombinant protein was analyzed by SDS/PAGE using an 15% polyacrylamide gel and confirmed by amino acid sequence analysis using a PPSQ‐31A protein sequencer (Shimadzu, Kyoto, Japan).

To obtain recombinant Cc‐PRI3(37–95) having an extra Gly‐Pro sequence at the *N*‐terminus (total 61 amino acids, calculated molecular weight 6398.18), recombinant 6 × His‐PRI3(37–95) was incubated with HRV‐3C protease (Japan Bioserum, Hiroshima, Japan) [30] in 20 mm HEPES‐NaOH (pH 7.0) at 4 °C for 24 h. The reaction mixture was applied to an XBridge Peptide BEH C18 (0.46 × 25 cm) column (Waters, Milford, MA, USA) and eluted with a linear gradient of 5%–50% acetonitrile containing 0.1% trifluoroacetic acid over 50 min at a flow rate of 0.8 mL·min^−1^, using an LC‐20 AD pump system (Shimadzu). The eluted recombinant Cc‐PRI3(37–95) was lyophilized *in vacuo*, dissolved in 20 mm sodium phosphate (pH 6.8), and confirmed by amino acid sequence analysis. The presence of free sulfhydryl groups in the recombinant protein was examined using Ellman's reagent [32]. In this assay, an aliquot of the recombinant protein (final concentration 4 μm) was incubated with 5,5′‐dithio‐*bis*‐(2‐nitrobenzoic acid) (0.2 mm; Fujifilm Wako Chemicals) in 100 mm phosphate buffer (pH 8.0) at room temperature for 15 min, and the absorbance of the solution at 412 nm was determined. Before use in the antimicrobial assay, the protein solution was sterilized by filtration using a 0.2‐μm pore‐size filter (Merck Millipore) and the protein concentration was determined spectrophotometrically using a molar extinction coefficient (*ε*) of 4170 m ^−1^ cm^−1^ at 280 nm.

### Fungal mycelial growth inhibition assay

Recombinant Cc‐PRI3(37–95) in 20 mm sodium phosphate buffer (pH 6.8) was mixed rapidly with potato dextrose agar (PDA) at final concentrations of 0.01–0.4 mg·mL^−1^ at 60 °C, and then 400 μL of PDA containing different concentrations of the recombinant protein was poured into the wells of a 12‐well plate. PDA containing only the buffer was employed in negative controls. One hundred conidia of *A*. *nidulans* FGSC A4 were point‐inoculated into the center of the medium and cultivated at 30 °C. Mycelia of *Alternaria porri* MAFF237561 or *F*. *oxysporum* f. sp. *cepae* 205 [26] that were grown in advance on PDA were clipped off in a circle with a diameter of 1 mm, inoculated into the center of the recombinant protein‐containing medium in an each well of the plate, and cultivated at 25 °C. The area of mycelial colonies that developed on the medium was recorded after 48 h for *A. nidulans* or *F. oxysporum*, and after 120 h for *Alternaria porri*. The IC_50_, which is the protein concentration required to reduce the area of the mycelial colony to 50% of that in the negative control, was determined from dose–response curves (percentage growth inhibition versus protein concentration) in triplicate experiments.

To assess the thermal stability of the antifungal activity, Cc‐PRI3(37–95) at a concentration of 1 mg·mL^−1^ in 50 mm Tris–HCl (pH 7.5) was kept at room temperature and 100 °C, respectively, for 10 min. To examine the significance of disulfide bridges, Cc‐PRI3(37–95) was also incubated with 5 mm dithiothreitol in 50 mm Tris–HCl (pH 7.5) at 100 °C for 10 min. PDA containing the heat‐treated or untreated Cc‐PRI3(37–95) at a concentration of 0.1 mg·mL^−1^ was prepared in a 12‐well plate and assay of inhibition of the mycelial growth of *A. nidulans* was performed as above. In the control, PDA medium containing the same amount of 5 mm dithiothreitol in 50 mm Tris–HCl (pH 7.5) was used. Each experiment was performed in triplicate.

To examine any effect of the amino acid sequence from Leu‐21 to Arg‐36 on the antifungal activity of recombinant Cc‐PRI3(37–95), a peptide with sequence LPPGPTSLEVEALEGR‐NH_2_ (calculated molecular weight 1663.87) was synthesized and confirmed by mass spectrometry by Eurofins Genomics. The synthetic peptide (130 μg) and the recombinant protein (50 μg) were preincubated at a molar ratio of 10 : 1 in 100 μL of 20 mm sodium phosphate buffer (pH 6.8) at room temperature for 30 min, and then infiltrated into 400 μL of PDA containing 3% (w/v) dry bouillon (Nissui, Tokyo, Japan) in each well of a 12‐well plate. PDA medium containing only the synthetic peptide was also prepared. One hundred conidia of *A. nidulans* were point‐inoculated into the center of the medium and cultivated at 30 °C. The area of mycelial colony developed on the medium was recorded; the experiment was performed in triplicate.

### Assay of inhibition of conidiogenesis of *A. nidulans*

One milliliter of PDA containing Cc‐PRI3(37–95) (0.1 mg·mL^−1^) was prepared in a 35 mm dish, as described for the fungal mycelial growth inhibition assay. Conidia of *A. nidulans* (1.5 × 10^4^) were spread across the medium surface and cultivated at 30 °C for 48 h. Newly generated conidia were collected by scraping the surface of the medium using a spreader and 0.5% Tween 20 aqueous solution, and then counted using a hemocytometer. Each experiment was performed in triplicate. Conidiophore morphology of *A. nidulans* grown on the PDA containing Cc‐PRI3(37–95) (0.1 mg·mL^−1^) was observed under an Olympus BX53 microscope equipped with phase‐contrast.

### Fluorescent staining of *A. nidulans*

Fluorescent staining of *A. nidulans* was performed as described [33] with minor modifications. Briefly, 1 × 10^3^ conidia of *A. nidulans* were cultivated in potato dextrose broth (PDB) with or without recombinant Cc‐PRI3(37–95) (0.1 mg·mL^−1^) at 30 °C overnight. An aliquot of the medium containing the hyphae of *A. nidulans* was dropped onto a slide glass, washed with PBS, incubated with solution containing 1 mg·mL^−1^ calcofluor‐white (CFW; Sigma‐Aldrich) and 0.5 mg·mL^−1^ Evans blue in the dark for 1 min, and then incubated with 10% (w/v) potassium hydroxide, before microscopic observation. The hyphae were also incubated with 0.32 mm carboxyfluorescein diacetate (CFDA) solution (Dojindo Laboratories, Kumamoto, Japan) in the dark at 36 °C for 5 min, with 1.4 μm propidium iodide (PI) solution (Dojindo Laboratories) in the dark at room temperature for 5 min, and then washed with PBS, before microscopic observation. The specimens were examined under an Olympus BX53 microscope equipped with phase‐contrast. Time‐lapse images of PI staining during the hyphal growth of *A. nidulans* were captured every 30 min for 24 h, in a medium containing 0.1 mg·mL^−1^ Cc‐PRI3(37–95), 3% (w/v) dry bouillon, 2% glucose and 1.4 μm PI, using a Keyence BZ‐X800 microscope (Keyence, Osaka, Japan).

### Leakage assay of intracellular materials

One hundred *A. nidulans* conidia were inoculated into 100 μL of a liquid medium containing 2% (w/v) glucose and 0.1% (w/v) dry bouillon and cultured at 30 °C for 40 h. The hyphae grown in the medium were washed twice with PBS, resuspended in 100 μL PBS containing recombinant Cc‐PRI3(37–95) at a final concentration of 0.1, 0.2, or 0.4 mg·mL^−1^, and then incubated at 30 °C for 5 h. As the control, the hyphae were incubated in 100 μL PBS without the recombinant protein. Nucleotides leaked from the hyphae were detected as described elsewhere [34] with some modifications. After centrifugation, an aliquot (20 μL) of the supernatant of the PBS medium was mixed with 180 μL QuantiFluor ONE dsDNA Dye (Promega, Madison, WI, USA), a fluorescent dye that binds selectively to double‐stranded DNAs over other nucleic acids. The fluorescence of the solution was measured using a Quantus Fluorometer (Promega) at excitation/emission wavelengths of 504/531 nm, respectively. As a standard, 20 μL lambda DNA (20 ng·μL^−1^) in PBS was used. Each experiment was performed in triplicate.

To detect protein leakage from the hyphae, an aliquot (30 μL) of the supernatant of the PBS medium was mixed with 120 μL working solution containing NanoOrange protein quantitation reagent (Molecular Probes, Eugene, OR, USA) that was diluted 500‐fold in NanoOrange protein quantitation diluent (Molecular Probes), and then incubated at 100 °C for 10 min. As a standard, 1 or 2 μg bovine serum albumin (Molecular Probes) dissolved in 30 μL of PBS was used. The fluorescence of samples in 96‐well plates was measured using a Fluoroskan FL plate reader (Thermo Fisher Scientific) at excitation/emission wavelengths of 485/538 nm, respectively. Each experiment was performed in triplicate.

### DPH‐binding assay

The membrane‐binding ability of 1,6‐diphenyl‐1,3,5‐hexatriene (DPH) was assayed as described elsewhere [34, 35, 36] with some modifications. One hundred *A. nidulans* conidia were inoculated into 100 μL of a liquid medium containing 2% (w/v) glucose and 0.1% (w/v) dry bouillon, and cultured at 30 °C overnight. Recombinant Cc‐PRI3(37–95) at a final concentration of 0.1, 0.2, or 0.4 mg·mL^−1^ was added to the hyphae‐containing medium before culturing at 30 °C overnight. As a control, the same amount of phosphate buffer (pH 6.8) was added to the hyphae before culturing at 30 °C overnight. After washing with PBS, the hyphae were incubated with 0.6 mm DPH (Sigma‐Aldrich) in PBS for 90 min. After washing twice with PBS, the samples were added to 96‐well plates, and the fluorescence intensity of DPH bound to the hyphal membrane was determined using a Fluoroskan Ascent FL plate reader (Thermo Fisher Scientific) at excitation/emission wavelengths of 355/460 nm, respectively. Each experiment was performed in triplicate.

### Antimicrobial assay of bacteria and yeasts

*Escherichia coli* DH5α or *M. luteus* was cultivated in 3% (w/v) dry bouillon, with or without recombinant Cc‐PRI3(37–95) (0.4 mg·mL^−1^), at 30 °C and 160 rpm for 24 h. The yeasts *Schizosaccharomyces japonicus* NRRL Y‐1026E, *Saccharomyces cerevisiae* sigma 1278b, *P. pastoris* X‐33, and *P. pastoris* SMD1168H were cultivated in YPD medium with or without the recombinant protein (0.4 mg·mL^−1^) at 30 °C and 700 rpm for 24 h. Absorbance at 620 nm was monitored every 2 h using a Multiskan FC plate reader (Thermo Fisher Scientific) and a 96‐well plate. Each experiment was performed in triplicate. Antimicrobial assays of the bacteria and yeasts were also performed on PDA medium containing 3% (w/v) dry bouillon in 100‐mm dishes. After inoculation of each bacterium or yeast onto solid medium, paper disks (8 mm in diameter) containing 200 μg of recombinant Cc‐PRI3(37–95) or buffer only (control) were placed on the medium. The dishes were incubated at 30 °C overnight, and the formation of a visible halo‐zone around the disk was examined.

### Statistical analysis

All experiments for statistical analysis were performed at least in triplicate. The data are represented as mean values ± SD (standard deviation). Statistical significance was determined using Student's *t*‐test. Significant differences between the control and samples are indicated by *P*‐values < 0.05 (*), < 0.01 (**), and < 0.001 (***).

## Results

### Isolation and characterization of a Pri3‐related cDNA from *C. cylindracea*

We obtained a *Pri3*‐related cDNA from *C. cylindracea* by reverse transcription‐PCR using an oligo‐dT primer and specific primers based on the genomic sequence of *C. cylindracea* [27] and the exon/intron boundaries in the untranslated 5′‐sequence of the *Ac‐Pri3* gene (AF369627) from *C. chaxingu* [21]. The cDNA contained an open reading frame that had the same nucleotide sequence as those of the genomic sequence of *C. cylindracea* and the *Ac‐Pri3* gene and is referred to here as *Cc‐Pri3* (Fig. S1). The *Cc‐Pri3* gene was predicted to encode a protein of 95 amino acids with a calculated molecular weight of 10 000.46 and a pI of 7.74 (Fig. 1). The Cc‐PRI3 protein was predicted to have an *N*‐terminal signal peptide of 20 amino acids using software based on the eukaryotic consensus sequence for signal peptidase cleavage sites [37], as similarly predicted for other proteins deduced from *Pri3* genes from *C. aegerita* [21]. The region from Leu‐21 to the final residue of the Cc‐PRI3 protein, Ala‐95, referred to as Cc‐PRI3(21–95), has a calculated molecular weight of 7890.84 and pI of 7.45. The *C*‐terminal region from Ala‐37 to Ala‐95, referred to as Cc‐PRI3(37–95), has a calculated molecular weight of 6244.02 and a high pI of 8.07 due to a wealth of basic residues; it is also rich in Gly residues and contains eight Cys residues at positions that are conserved among PRI3 proteins [21] (Fig. 1). In contrast, Cc‐PRI3(21–36), the residues from Leu‐21 to Arg‐36, has a low calculated pI value of 4.01. The *N*‐terminal amino acid sequence of Cc‐PRI3(37–95) is homologous to the *N*‐terminal 15 amino acid residues of agrocybin, an antifungal protein purified from the fruiting body of *C. cylindracea* [22] (Fig. 1).

### Fruiting body‐specific expression of the *Cc‐Pri3* gene

**Fig. 2:**
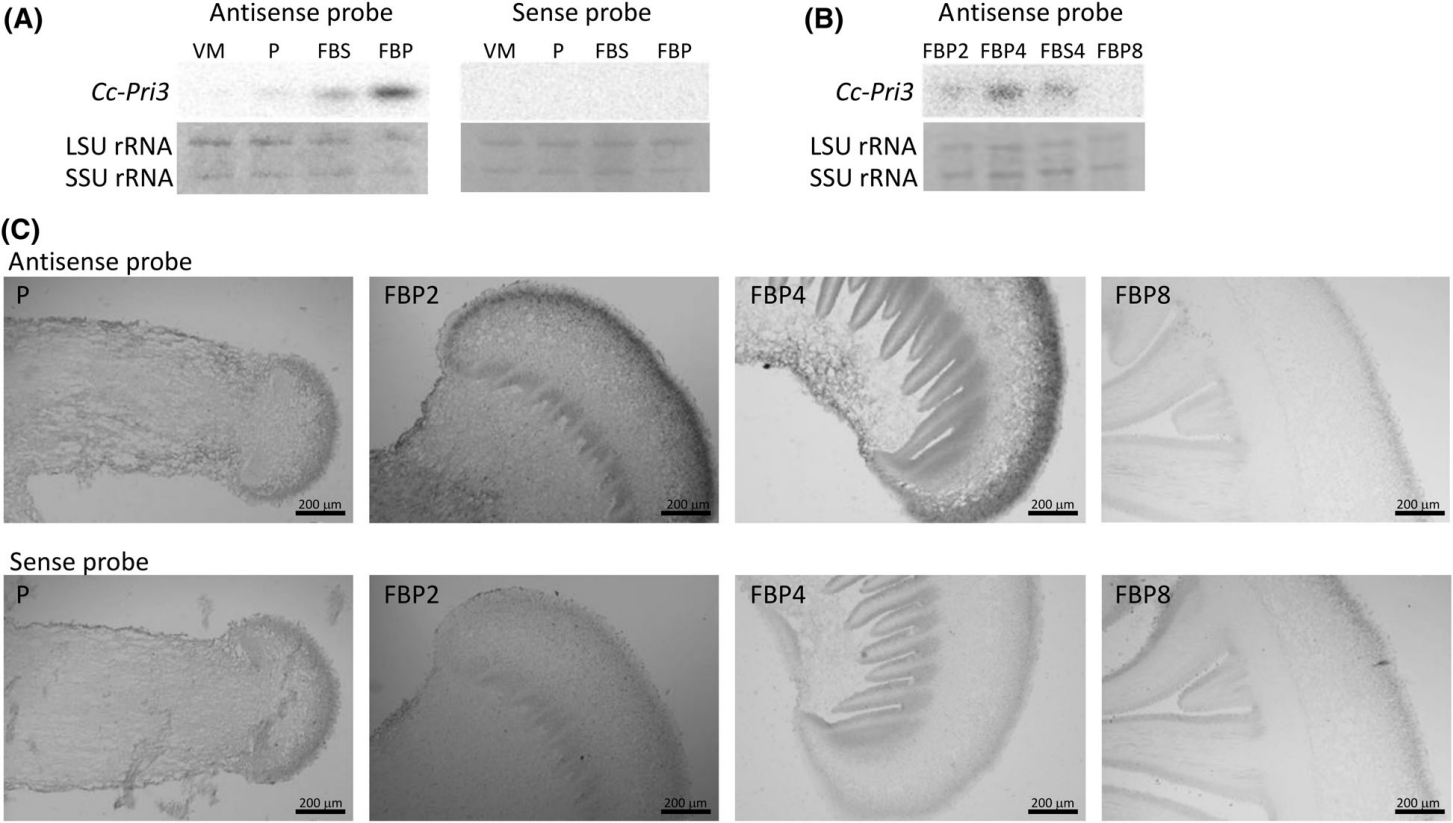
Fruiting body‐specific expression of *Cc‐Pri3* mRNA. (A) Northern blotting analysis of *Cc‐Pri3* mRNA from *C. cylindracea*. Total RNA was extracted from vegetative mycelia (abbreviated as VM), primordia (P), fruiting body stem (FBS), or fruiting body pileus (FBP), separated by gel electrophoresis, blotted, and probed with DIG‐labeled antisense or sense RNA probes (*n* = 3). (B) Similarly, total RNA was extracted from pileus of fruiting body of 2‐cm height (FBP2), pileus of fruiting body of 4‐cm height (FBP4), stem of fruiting body of 4‐cm height (FBS4), or pileus of fruiting body of 8‐cm height (FBP8) and analyzed using DIG‐labeled antisense probe (*n* = 3). The *Cc*‐*Pri3* gene is expressed primarily in the pileus of fruiting body of 4‐cm (FBP4), and slightly in the pileus of fruiting body of 2‐cm (FBP2) and in the stem of fruiting body of 4‐cm (FBS4), but not in the fruiting body of 8‐cm (FBP8). The small subunit (SSU) and large subunit (LSU) rRNAs stained with ethidium bromide are shown as loading controls. (C) *In situ* hybridization analysis of *Cc‐Pri3* mRNA. A thin section of primordia (P), or pileus of fruiting body of 2‐cm height (FBP2), 4‐cm height (FBP4), or 8‐cm height (FBP8) was probed using DIG‐labeled antisense or sense probe (*n* = 2, representative images shown). The *Cc*‐*Pri3* gene is expressed specifically in cells located close to the surface of the pileus of the fruiting body of 2 or 4 cm (FBP2 or FBP4), but not in primordia (P) or fruiting body of 8 cm (FBP8). Scale bars: 200 μm.

To confirm the expression site of the *Cc‐Pri3* gene in *C. cylindracea*, northern blotting analysis was performed for RNA samples obtained from the mycelia, primordia, fruiting body pileus, and stem of *C. cylindracea*, using DIG‐labeled antisense and sense RNA probes that correspond to the nucleotide positions from 154 to 436 of the *Cc‐Pri3* gene in Fig. S1. The results indicate that the *Cc*‐*Pri3* gene is expressed principally in the fruiting body pileus, but not in the mycelia or in the primordia of *C. cylindracea* (Fig. 2A). To determine the expression stage during fruiting body development, RNA samples extracted from the pileus and stem of fruiting bodies with heights of about 2, 4, and 8 cm were analyzed (Fig. 2B). We found that the *Cc*‐*Pri3* gene was expressed primarily in the pileus of immature fruiting bodies (4 cm height). We also detected *Cc*‐*Pri3* expression at low levels in the pileus of fruiting bodies of 2 cm height and in the stems of fruiting bodies of 4 cm height, but not in mature fruiting bodies (8 cm height) (Fig. 2B). These results indicate that, in *C. cylindracea*, the *Cc*‐*Pri3* gene is expressed specifically in the pileus of immature fruiting bodies with a height of 2–4 cm. In contrast, the *Aa1*‐*Pri3* gene of *C. aegerita* was reported to be specifically expressed in primordia [21].

*In situ* hybridization was performed using thin sections of the primordia and the pileus of fruiting bodies with heights of about 2, 4, and 8 cm, and the DIG‐labeled RNA probes (Fig. 2C). We found that the *Cc*‐*Pri3* gene is expressed specifically in cells located close to the surface of the pileus of the fruiting bodies with heights of 2 and 4 cm.

### Detection of native Cc‐PRI3 protein in fruiting body

**Fig. 3:**
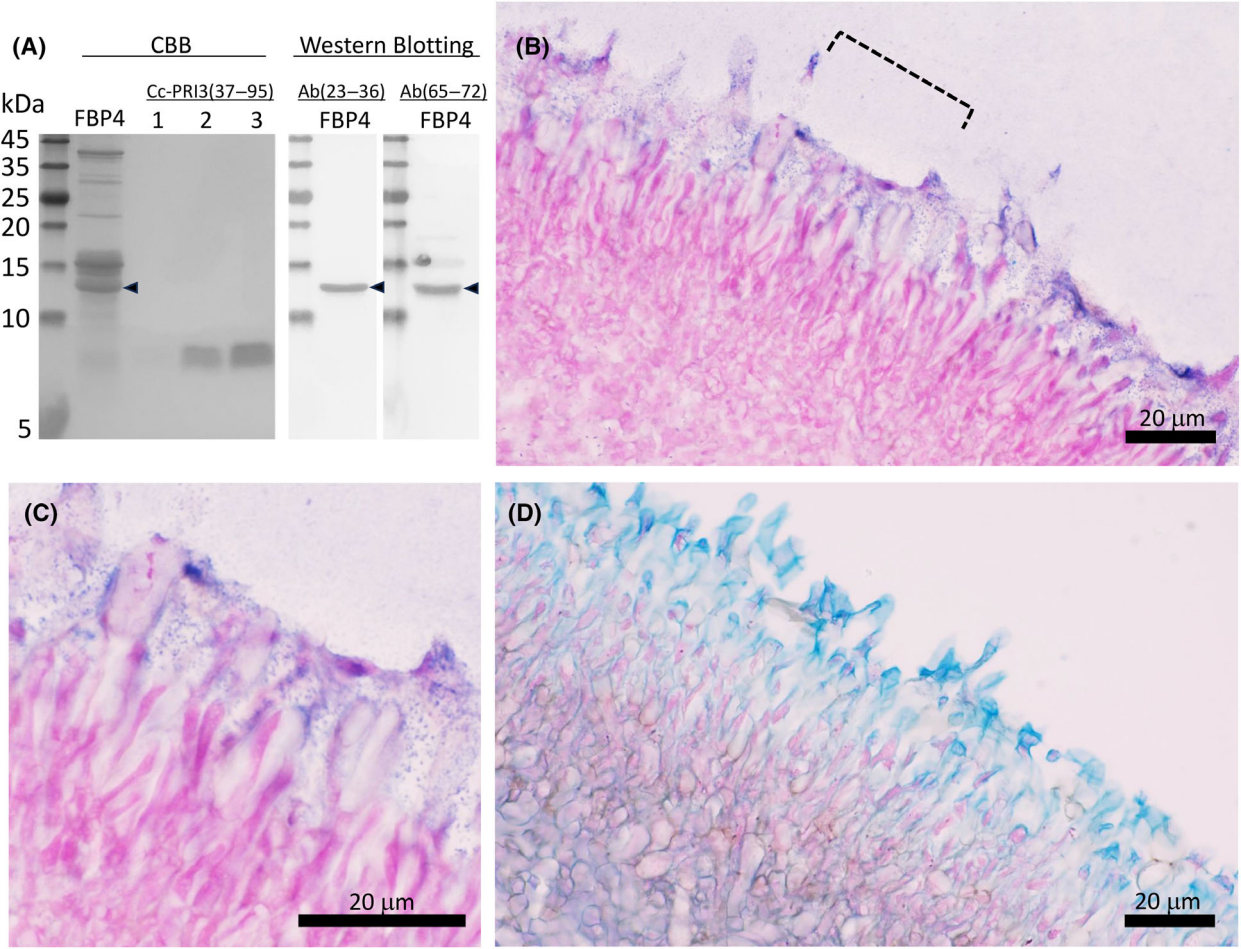
Western blotting and immunohistochemical analysis of native Cc‐PRI3 protein in fruiting body. (A) Lysate of the pileus of fruiting body of about 4‐cm height (FBP4) was centrifuged, and then the supernatant was precipitated stepwise by 40% ammonium sulfate and 80% ammonium sulfate. The proteins precipitated by 80% ammonium sulfate were heat‐treated in sample buffer containing 5 mm dithiothreitol and resolved by 18% SDS/PAGE (lanes: FBP4), transferred onto a membrane, and then detected using rabbit antiserum raised against a synthetic peptide representing residues 23–36 [Ab(23–36)] or residues 65–72 [Ab(65–72)] of the Cc‐PRI3 protein. Native Cc‐PRI3 (arrowhead) was detected as a band of 13 kDa using Coomassie Brilliant Blue (CBB) staining and both antisera (*n* = 2). The precipitates obtained using 40% ammonium sulfate showed no immunoreactive band (data not shown). Recombinant protein Cc‐PRI3(37–95) (lane 1, 0.1 μg; lane 2, 0.5 μg; lane 3, 1.0 μg) prepared in this study was used as a reference, and was detected as a band of 8.5 kDa by CBB staining (*n* = 2). (B) Immunohistochemical analysis using a thin section of the pileus of fruiting body of about 4‐cm height and the antiserum Ab(23–36) raised against the synthetic peptide representing residues 23–36 of Cc‐PRI3. Immunostaining with nitro blue tetrazolium and 5‐bromo‐4‐chrolo‐3‐indoyl phosphate (blue) and cell staining with Nuclear Fast Red solution (red) are shown (*n* = 3, representative images shown). Scale bar: 20 μm. The area indicated by the broken line is enlarged in panel C. Immunostaining was observed with a dot‐like pattern. Scale bar: 20 μm. (D) Thin section of the pileus of fruiting body of about 4‐cm height stained with Alcian blue solution (blue), which is specific for acidic polysaccharides, and with Nuclear Fast Red solution (red) (*n* = 2, representative images shown). Scale bar: 20 μm.

To detect native Cc‐PRI3 protein in the pileus of *C. cylindracea*, western blotting was performed using two antisera raised against synthetic peptides, respectively representing the residues from Pro‐23 to Arg‐36 and from Gln‐65 to Arg‐72 of the Cc‐PRI3 protein (Fig. 1). The latter region is in the *C*‐terminal Cys‐rich region of the protein, and the former is located between the *N*‐terminal signal peptide and the *C*‐terminal Cys‐rich region. Both antisera detected native Cc‐PRI3 protein as a band of 13 kDa in the pileus lysate from fruiting bodies of about 4 cm height (Fig. 3A). Other immunoreactive bands with a lower molecular weight, such as a band corresponding to the recombinant Cc‐PRI3(37–95) prepared in this study and shown as a reference in Fig. 3A, were not observed. The antiserum raised against the peptide composed of residues Gln‐65 to Arg‐72 detected the Cc‐PRI3 protein only when the protein was heat‐treated in SDS/PAGE sample buffer containing 5 mm dithiothreitol; without this treatment, the antiserum failed to detect the protein. This is probably due to a unique steric structure formed by the disulfide bridges in the *C*‐terminal Cys‐rich region of the native protein.

To localize the native Cc‐PRI3 protein in the pileus, immunohistochemical analysis was performed using sections of a fruiting body of about 4 cm height and the antiserum raised against the peptide representing residues Pro‐23 to Arg‐36 of the Cc‐PRI3 protein. The results indicate that native Cc‐PRI3 protein is secreted from the expressing cells into the outermost layer of the pileus, as detected in a dot‐like pattern (Fig. 3B,C). The reason for the dot‐like staining pattern is currently unknown. The outermost layer of the pileus also stained with Alcian blue solution [29], which indicates that the layer contains acidic polysaccharides (Fig. 3D).

### Preparation of recombinant Cc‐PRI3 protein

To help characterize the biological function of the Cc‐PRI3 protein, we intended to prepare recombinant protein using a *Pichia* expression system. At first, we attempted to generate the recombinant protein referred to as Cc‐PRI3(21–95)‐Myc‐6 × His (101 amino acids, calculated molecular weight 10 875.01) (Fig. S2A) using the expression vector pPICZαA [31] and *P. pastoris* X‐33 cells. The recombinant protein represents the residues from Leu‐21 to final Ala‐95 of Cc‐PRI3 and is tagged at the *C*‐terminus with Myc‐ and 6 × His‐tag sequences. However, the expression system gave two recombinant protein products, as shown in SDS/PAGE analysis of the sample that was obtained after His‐tag affinity column chromatography of the culture medium (Fig. S2B). Amino acid sequence analysis of the proteins indicated that about half of the recombinant Cc‐PRI3(21–95)‐Myc‐6 × His protein was truncated at the *C*‐terminal side of Arg‐36 by an unknown proteolytic enzyme. Consequently, the product giving the lower molecular weight band in SDS/PAGE (Fig. S2B), referred to as Cc‐PRI3(37–95)‐Myc‐6 × His (85 amino acids, calculated molecular weight 9228.19) (Fig. S2A), has the *N*‐terminal 15 residues that are homologous to those of agrocybin [22] (Fig. 1).

**Fig. 4:**
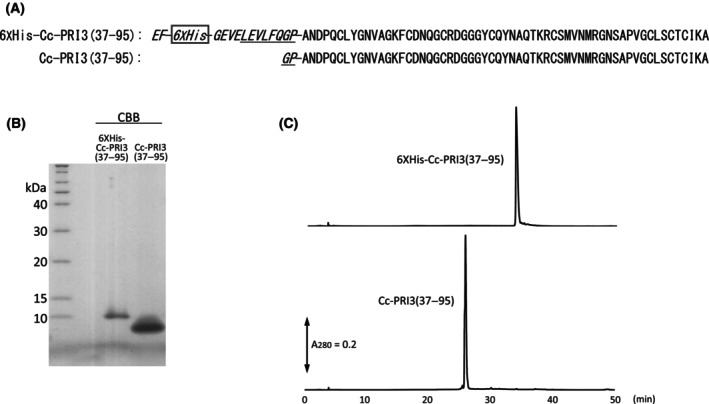
Preparation of recombinant Cc‐PRI3(37–95). (A) Amino acid sequences of recombinant 6 × His‐Cc‐PRI3(37–95) and Cc‐PRI3(37–95). Recombinant 6 × His‐Cc‐PRI3(37–95) has an extra *N*‐terminal sequence (marked in italics) of Glu‐Phe that is followed by a 6 × His‐tag sequence and the HRV‐3C protease recognition sequence (underlined). In the protein expression in *Pichia pastoris* SMD1168H cells, 6 × His‐Cc‐PRI3(37–95) was secreted into the yeast culture medium with the aid of the *N*‐terminal α‐factor sequence that is encoded in the expression vector and was removed during secretion into the medium. Cc‐PRI3(37–95) contains an extra Gly‐Pro *N*‐terminal sequence (marked in italics) that is left after the proteolysis by HRV‐3C protease. (B) SDS/PAGE analysis of recombinant 6 × His‐Cc‐PRI3(37–95) and Cc‐PRI3(37–95) (*n* = 2). The recombinant 6 × His‐Cc‐PRI3(37–95) in the culture medium was purified by His‐tag affinity column chromatography. Cc‐PRI3(37–95) was obtained from 6 × His‐Cc‐PRI3(37–95) by treatment with HRV‐3C protease, which cleaves the peptide bond between Gln and Gly in the recognition sequence. The protein bands were detected using CBB. (C) High‐performance liquid chromatography of 6 × His‐Cc‐PRI3(37–95) and Cc‐PRI3(37–95) (*n* = 2). Each recombinant protein was applied to a reverse‐phase column. Absorbance at 280 nm (A_280_) was recorded.

To avoid the proteolytic processing in the protein expression in *Pichia*, we redesigned a recombinant protein, referred to as 6 × His‐Cc‐PRI3(37–95) (79 amino acids, calculated molecular weight 8641.56, pI 6.63), which has an *N*‐terminal His‐tag sequence followed by the HRV‐3C protease recognition sequence (Leu‐Glu‐Val‐Leu‐Phe‐Gln‐Gly‐Pro) [30], then the Cc‐PRI3 sequence representing the residues from Ala‐37 to final Ala‐95 (Fig. 4A). The corresponding DNA fragment was ligated into vector pPICZαA and introduced into *P. pastoris* SMD1168H cells that lack the *pep4* gene, which encodes proteinase A [31]. SDS/PAGE analysis after His‐tag affinity column chromatography of the culture medium indicated that the recombinant protein was secreted into the yeast culture medium and gave a single band of 10 kDa (Fig. 4B). Then, the *N*‐terminal His‐tag sequence was removed by incubation with HRV‐3C protease, which cleaves the peptide bond between Gln and Gly in the Leu‐Glu‐Val‐Leu‐Phe‐Gln‐Gly‐Pro recognition sequence [30]. The resultant protein was detected as a single band of 8.5 kDa in SDS/PAGE analysis (Figs 3A and 4B), and was finally purified by reverse‐phase high‐performance liquid chromatography (Fig. 4C). Amino acid sequence analysis showed that the purified recombinant protein had the expected *N*‐terminal sequence, Gly‐Pro (remaining from the HRV‐3C protease recognition sequence), followed by the Cc‐PRI3(37–95) sequence (61 amino acids, calculated molecular weight 6398.18, pI 8.07) (data not shown). The apparent molecular weight of the recombinant Cc‐PRI3(37–95) detected in SDS/PAGE analysis was close to that reported for agrocybin (9 kDa) [22]. The results of the Ellman's assay [32] showed that there were no free sulfhydryl groups in the recombinant Cc‐PRI3(37–95), suggesting that four disulfide bridges were present in the recombinant protein.

### Antifungal activity of recombinant Cc‐PRI3 protein

**Fig. 5:**
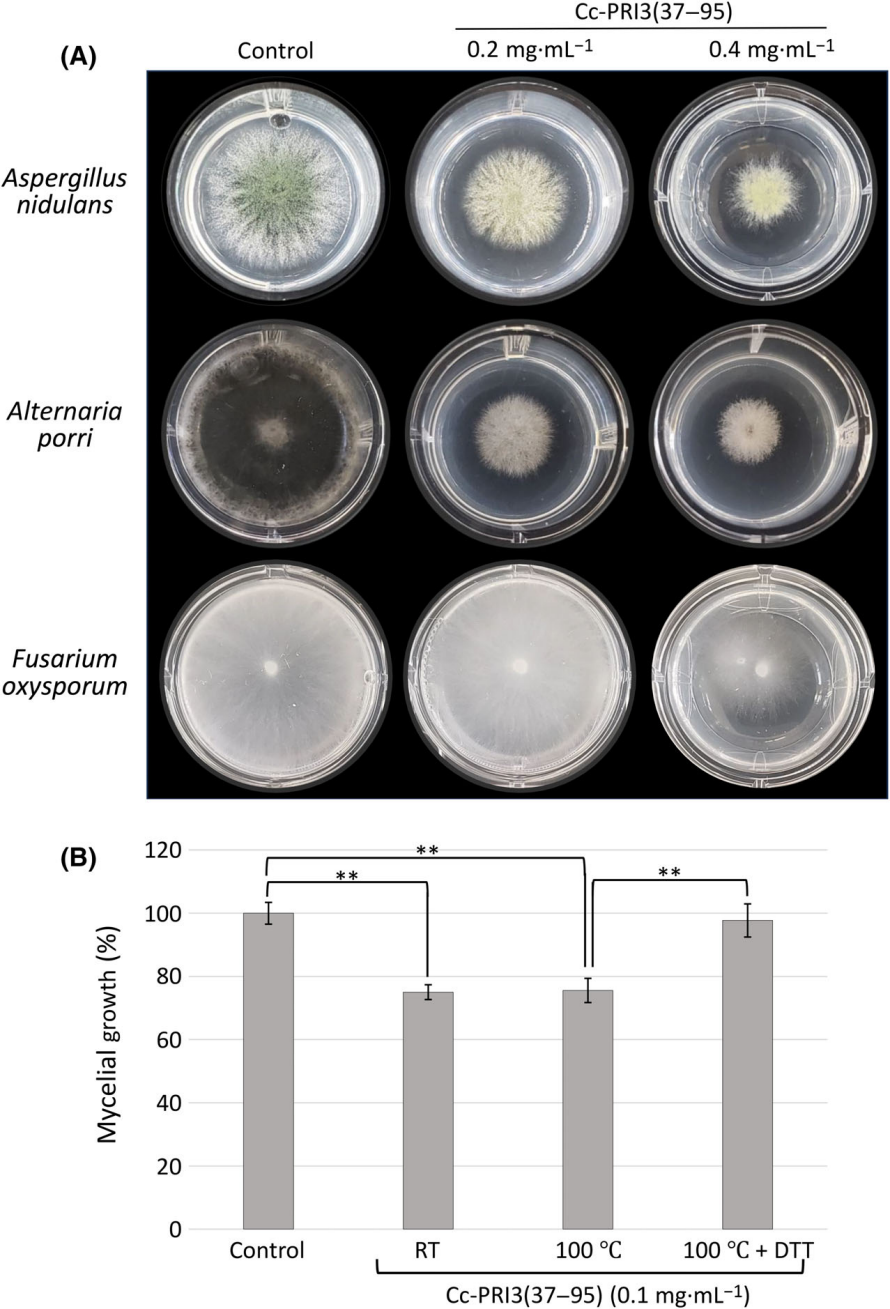
Inhibition of fungal mycelial growth by recombinant Cc‐PRI3(37–95). (A) Mycelia of *Aspergillus nidulans*, *Alternaria porri*, and *Fusarium oxysporum* were cultivated on potato dextrose agar (PDA) containing recombinant Cc‐PRI3(37–95) at final concentrations of 0.01–0.4 mg·mL^−1^. The area of mycelial colonies developed on the medium was recorded (*n* = 3). Typical images for 0.2 and 0.4 mg·mL^−1^ Cc‐PRI3(37–95) are shown. The recombinant protein inhibited mycelial growth of the fungi. (B) Thermal stability of the antifungal activity of recombinant Cc‐PRI3(37–95). The recombinant protein in 50 mm Tris–HCl (pH 7.5) was kept at room temperature (RT) or 100 °C for 10 min and then tested in the *A*. *nidulans* mycelial growth inhibition assay at a final concentration of 0.1 mg·mL^−1^. The antifungal activity of the recombinant protein was maintained even after treatment at 100 °C for 10 min, but was diminished by treatment with 5 mm dithiothreitol (DTT) in 50 mm Tris–HCl (pH 7.5) at 100 °C for 10 min. In the control, the same amount of 5 mm dithiothreitol in 50 mm Tris–HCl (pH 7.5) was added to PDA medium. Each experiment was performed in triplicate, and all data are displayed as mean values ± SD. Statistically significant differences from control were determined using Student's *t*‐test and indicated as **P < 0.01.

To help characterize the function of Cc‐PRI3, the antifungal activity of recombinant Cc‐PRI3(37–95) was determined against three kinds of fungi. *Aspergillus nidulans* FGSC A4 is a model filamentous fungus, and *Alternaria porri* MAFF237561 and *Fusarium oxysporum* f. sp. *cepae* 205 are plant pathogens that cause onion purple blotch and onion dry rot, respectively [26]. As shown in Fig. *5*A, the recombinant Cc‐PRI3(37–95) inhibited mycelial growth of these fungi. The IC_50_ values were determined to be 0.13 mg·mL^−1^ (20 μm) for *A. nidulans*, 0.03 mg·mL^−1^ (5 μm) for *Alternaria porri*, and 0.25 mg·mL^−1^ (39 μm) for *F. oxysporum* (Table 1). *Thus*, the recombinant Cc‐PRI3(37–95) exhibited efficient inhibition of the growth of the three fungi tested here. *T*he antifungal activity of recombinant Cc‐PRI3(37–95) against *A. nidulans* was observed to be stable even after treatment of the recombinant protein at 100 °C for 10 min, whereas the antifungal activity was extinguished by heat treatment in the presence of 5 mm dithiothreitol (Fig. 5B), suggesting that disulfide bridges in the recombinant Cc‐PRI3(37–95) are critical for the antifungal activity.

**Table 1:** Growth inhibitory activity of recombinant Cc‐PRI3(37–95) against three fungi. Protein concentrations required for 50% growth inhibition (IC_50_) were determined from the dose–response curves (percentage growth inhibition versus protein concentration). Growth of *Aspergillus nidulans* or *Fusarium oxysporum* was measured after 48 h incubation, while growth of slowly growing *Alternaria porri* was measured after 120 h incubation.

Fungus	IC_50_, mg·mL^−1^ (μm)
*Aspergillus nidulans* FGSC A4	0.13 (20)
*Alternaria porri* MAFF237561	0.03 (5)
*Fusarium oxysporum* f. sp. *cepae* 205	0.25 (39)

The residues from Leu‐21 to Arg‐36 of Cc‐PRI3 are acidic (pI 4.01) and might play a role in regulating the antifungal activity of the *C*‐terminal Cys‐rich region, Cc‐PRI3(37–95) (pI 8.07). To test this hypothesis, recombinant Cc‐PRI3(37–95) was preincubated with a synthetic peptide, Leu‐Pro‐Pro‐Gly‐Pro‐Thr‐Ser‐Leu‐Glu‐Val‐Glu‐Ala‐Leu‐Glu‐Gly‐Arg‐NH_2_, representing residues Leu‐21 to Arg‐36 of Cc‐PRI3, at a molar ratio of 1 : 10, and then, the mixture was used in antimicrobial assay against *A. nidulans*. No significant effect of the peptide was observed (data not shown), suggesting that the residues from Leu‐21 to Arg‐36 are not involved in the antifungal activity of Cc‐PRI3(37–95). The synthetic peptide itself showed no antifungal activity (data not shown).

**Fig. 6:**
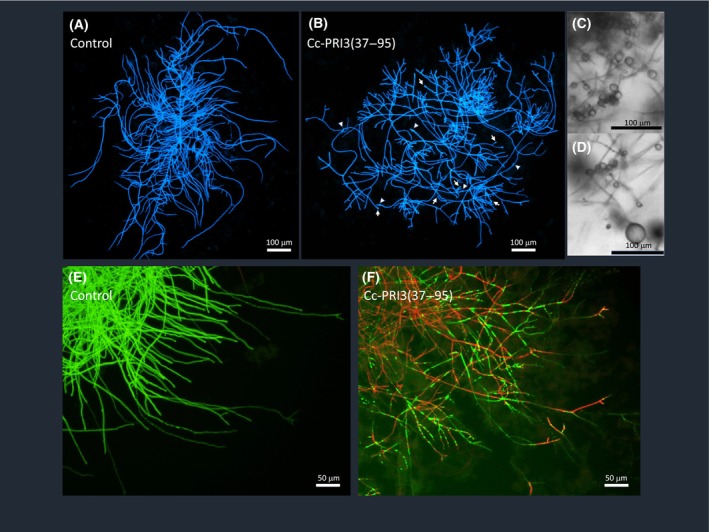
Effects of recombinant Cc‐PRI3(37–95) on the morphology of *A*. *nidulans*. Conidia of *A. nidulans* were cultivated in potato dextrose broth without (A, E: control) or with (B, F) recombinant Cc‐PRI3(37–95) at 0.1 mg·mL^−1^. An aliquot of medium containing hyphae was dropped on a slide glass, and incubated with calcofluor‐white (A, B) or with carboxyfluorescein diacetate (CFDA, green) and propidium iodide (PI, red) (E, F) before fluorescence microscopic observation. (A, B, E, F: *n* = 2, representative images shown). In (B), hyphal branching is increased compared with that in the control (A). Hyphal tip swelling (indicated by arrows) and balloon formation (indicated by arrowheads) were also observed. Both (C) and (D) Balloon formation was frequently observed by phase‐contrast microscopy when conidia of *A. nidulans* were cultivated in potato dextrose agar containing recombinant Cc‐PRI3(37–95) at 0.1 mg·mL^−1^ (C, D: *n* = 2, representative images shown). (E) Hyphal cells in the control were stained only by CFDA (green). (F) Hyphal cells grown in medium containing recombinant Cc‐PRI3(37–95) were stained extensively by PI (red) and partly by CFDA (green). PI‐ and CFDA‐stained cells were observed in a mosaic pattern along the same hypha. Scale bars: 100 μm (A–D), 50 μm (E, F).

Morphological effects of recombinant Cc‐PRI3(37–95) on hyphae of *A. nidulans* were visualized microscopically (Fig. 6). Corresponding to the mycelial growth inhibition, chitin staining of *A. nidulans* with CFW revealed that the recombinant Cc‐PRI3(37–95) increased hyphal branching, tip swelling, and balloon formation (Fig. 6B), when compared with control (Fig. 6A). Balloon formation by the recombinant protein was more frequently observed when *A. nidulans* was cultivated on PDA (Fig. 6C,D: indicated in phase‐contrast microscopy) rather than in PDB (Fig. 6B). Co‐staining of the hyphae with CFDA and PI revealed that the recombinant protein significantly decreased the viability of hyphal cells (Fig. 6E,F). CFDA permeates into the cytoplasm through cell membranes and is hydrolyzed to the fluorescent carboxyfluorescein by intracellular esterases in live cells [33, 38]. PI is a membrane‐impermeable dye, but it can enter cells with a compromised membrane and bind to nucleotides to emit fluorescence [33, 39]. While the hyphal cells in the control were stained only by CFDA (Fig. 6E), the hyphal cells grown in medium containing recombinant Cc‐PRI3(37–95) stained extensively with PI and partly with CFDA (Fig. 6F). This result indicates that the recombinant protein increased the permeability of the plasma membrane and decreased the viability of the hyphal cells. Interestingly, PI‐ and CFDA‐stained cells were observed in a mosaic pattern along the same hypha (Fig. 6F). Time‐lapse images of the PI‐staining pattern during the hyphal growth can be seen in Supporting Information (Video S1). After hyphae elongated to some extent, the PI‐staining started in different cells along the same hypha with a lag‐time.

**Fig. 7:**
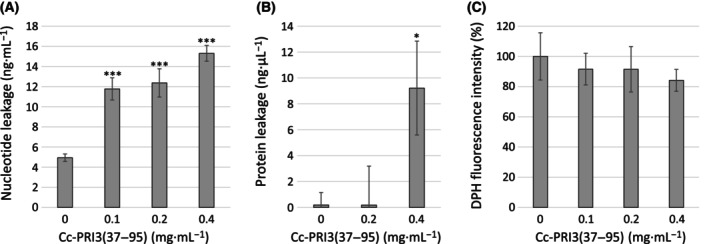
Assessment of membrane integrity of *A. nidulans* hyphae. (A) *A. nidulans* hyphae suspended in PBS were treated with recombinant Cc‐PRI3(37–95) (0, 0.1, 0.2, 0.4 mg·mL^−1^) at 30 °C for 5 h, and centrifuged. Supernatants were analyzed to determine concentrations of nucleotides leaked from hyphae using a fluorescent dye that binds selectively to double‐stranded DNAs over other nucleic acids. Each experiment was performed in triplicate, and all data are displayed as mean values ± SD. Statistically significant differences from control were determined using Student's *t*‐test and indicated as ***P < 0.001. (B) Similarly to (A), *A. nidulans* hyphae suspended in PBS were treated with the recombinant protein (0, 0.2, 0.4 mg·mL^−1^) at 30 °C for 5 h, and centrifuged. Supernatants were analyzed to determine concentrations of proteins leaked from the hyphae, using a fluorescent dye that binds selectively to proteins. Each experiment was performed in triplicate, and all data are displayed as mean values ± SD. Statistically significant difference from control was determined using Student's *t*‐test and indicated as *P < 0.05. (C) *A. nidulans* hyphae in liquid medium (2% glucose and 0.1% dry bouillon; w/v) were treated with the recombinant protein (0, 0.1, 0.2, 0.4 mg·mL^−1^) at 30 °C overnight, washed with PBS, and then incubated with 0.6 mm 1,6‐diphenyl‐1,3,5‐hexatriene (DPH). After washing with PBS, relative fluorescence intensity of DPH in the hyphae was assessed. Each experiment was performed in triplicate, and all data are displayed as mean values ± SD. The DPH fluorescence intensity decreased, as the concentration of the recombinant protein increased, although statistically significant differences (*P* < 0.05) from control were not observed using Student's *t*‐test.

To explore the reason for the increase in plasma membrane permeability, we examined whether Cc‐PRI3(37–95) causes leakage of intracellular materials from *A. nidulans* hyphal cells. The hyphae suspended in PBS were treated with the recombinant protein at different concentrations for 5 h, and then, the supernatant was analyzed to detect nucleotides and proteins leaked from the hyphal cells. These analyses were conducted using two kinds of fluorescent dyes that selectively bind to double‐stranded DNAs and proteins. As shown in Fig. 7A, the recombinant protein at concentrations of 0.1–0.4 mg·mL^−1^ increased nucleotide leakage from the hyphal cells, in a concentration‐dependent manner. Figure 7B shows that the recombinant protein at the concentration of 0.4 mg·mL^−1^ caused substantial protein leakage. These results suggest that Cc‐PRI3(37–95) disrupted the hyphal cell membranes, leading to the leakage of intracellular nucleotides and proteins.

To further characterize the effect of recombinant Cc‐PRI3(37–95) on hyphal cell membranes, DPH was used as a probe to monitor changes in the plasma membrane integrity. DPH is almost non‐fluorescent in water, but it shows strong fluorescence after intercalation into the membrane lipid bilayer [34, 35, 36]. If the recombinant protein disrupts the membrane lipid bilayer, the intercalation of DPH is reduced, resulting in a decrease in DPH fluorescence intensity [34, 35, 36]. As shown in Fig. 7C, recombinant Cc‐PRI3(37–95) at the concentrations of 0.1–0.4 mg·mL^−1^ decreased the DPH fluorescence intensity of the *A. nidulans* hyphal cells, compared with that of the control, and this effect was concentration‐dependent. These results support the view that the recombinant protein disrupted the membrane lipid bilayer of the hyphal cells.

**Fig. 8:**
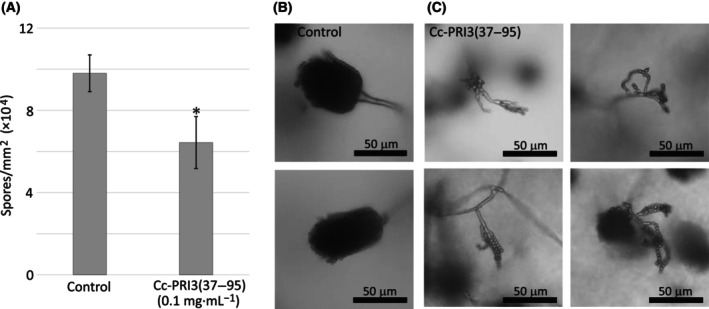
Inhibition of conidiogenesis of *A. nidulans* by recombinant Cc‐PRI3(37–95). (A) Recombinant Cc‐PRI3(37–95) at 0.1 mg·mL^−1^ in PDA decreased the number of newly formed spores of *A. nidulans* to about 60% of that in the control. Each experiment was performed in triplicate, and the data are displayed as mean values ± SD. Statistically significant difference from control was determined using Student's *t*‐test and indicated as *P < 0.05. (B, C) Compared with the control (B: 20 mm sodium phosphate buffer only; two representative images shown), recombinant Cc‐PRI3(37–95) at 0.1 mg·mL^−1^ in PDA caused abnormal conidiophore morphologies (C, four representative images shown). Scale bars: 50 μm.

The recombinant Cc‐PRI3(37–95) also inhibited conidiogenesis of *A. nidulans*, as shown in Fig. 8A. The recombinant protein at 0.1 mg·mL^−1^ in PDA decreased the number of newly formed spores of *A. nidulans* to about 60% of that in the control. Recombinant Cc‐PRI3(37–95) in PDA also caused abnormal conidiophore morphologies, as compared with conidiophores in the control (Fig. 8B). Typical micrographs are shown in Fig. 8C.

Recombinant Cc‐PRI3(37–95) did not inhibit proliferation of the Gram‐negative bacterium *E. coli* DH5α, the Gram‐positive bacterium *M. luteus*, the fission yeast *Schizosaccharomyces japonicus* NRRL Y‐1026E, or the budding yeast *Saccharomyces cerevisiae* sigma1278b, *P. pastoris* X‐33, and *P. pastoris* SMD1168H in liquid medium [3% (w/v) dry bouillon or YPD medium, Fig. S3] or on solid medium [PDA containing 3% (w/v) dry bouillon, Fig. S4]. Recombinant Cc‐PRI3(37–95) did not inhibit mycelial growth of *C*. *cylindracea* itself on PDA medium (data not shown).

## Discussion

In this study, a *Pri3*‐related gene, *Cc‐Pri3*, was cloned from *C. cylindracea*, and was observed to be specifically expressed at the pileus surface of immature fruiting bodies, although the *Aa1‐Pri3* gene was previously reported to be specifically expressed in the primordia of *C. aegerita* [21]. The reason for the difference in expression pattern between the two genes is unknown, although there is a possibility that biogeographic separation between *C. cylindracea* (Asia) and *C. aegerita* (Europa) might affect the gene expression site and stage during fruiting body development. While immature fruiting bodies of *C. cylindracea* perform successively stipe elongation and pileus maturation including gill development and spore formation [4], the expression of the *Cc‐Pri3* gene seems to be related to pileus maturation.

The *Cc‐Pri3* gene encodes a small Cys‐rich protein, referred to as Cc‐PRI3, which is predicted to have an *N*‐terminal signal peptide of 20 amino acids, as also predicted for other *Pri3* gene products from *C. aegerita* [21]. Immunohistochemical analysis revealed that native Cc‐PRI3 protein is actually secreted from the expressing cells located close to the pileus surface into the outermost layer of the pileus, which contains acidic polysaccharides. In the western blotting analysis of the pileus lysate, native Cc‐PRI3 protein was detected as a band of 13 kDa by antisera raised against synthetic peptides respectively representing the residues from Pro‐23 to Arg‐36 and from Gln‐65 to Arg‐72 of the Cc‐PRI3 protein. Other immunoreactive bands with lower molecular weight, such as a band corresponding to the recombinant Cc‐PRI3(37–95), were not observed. These results suggest that native Cc‐PRI3 protein exists in the form of Cc‐PRI3(21–95) in the polysaccharide layer, without further proteolytic processing after the cleavage of the 20‐residue *N*‐terminal signal peptide. The band detected in the western blotting analysis showed a slightly higher molecular weight than the calculated weight (7890.84) of Cc‐PRI3(21–95); this might be partly due to glycosylation. The Thr‐26 residue in the sequence of Pro ^23^‐Gly^24^‐Pro ^25^‐*Thr* ^26^‐Ser ^27^ is a candidate site for *O*‐glycosylation, because Pro at the −1 and − 3 positions and Ser at the +1 position constitute a sequence motif that favors *O*‐glycosylation [40]. The dot‐like staining pattern observed in the immunohistochemical analysis raises the possibility that the native Cc‐PRI3 protein might exist in homopolymeric complexes or as a component in exosomes or exosome‐like particles; such nanoparticles have been isolated and characterized for several mushrooms [41] but not yet for *C. cylindracea*.

In the preparation of recombinant Cc‐PRI3(21–95)‐Myc‐6 × His protein, proteolytic cleavage of the peptide bond between residues Arg‐36 and Ala‐37 was observed. Some protease activities that truncate recombinant proteins have been described for intracellular vacuoles and fermentation culture supernatant of *Pichia*. These include Kex2 [42], YPS1 [43], proteinase A [44] and proteinase B [45, 46]. Considering the substrate specificities, proteinase B is a candidate for the protease that cleaved the recombinant Cc‐PRI3(21–95)‐Myc‐6 × His: proteinase B is a subtilisin‐like serine endopeptidase with fairly broad substrate specificity similar to chymotrypsin and trypsin [45, 46]. The conversion of recombinant Cc‐PRI3(21–95)‐Myc‐6 × His to Cc‐PRI3(37–95)‐Myc‐6 × His in the *Pichia* expression system raises the possibility that a similar proteolytic processing that cleaves at the *C*‐terminal side of Arg‐36 might occur in native Cc‐PRI3 protein during the biosynthesis of the protein in the pileus of *C. cylindracea*, which would give Cc‐PRI3(37–95), having the *N*‐terminal sequence homologous to that reported for agrocybin [22]. However, our western blotting and immunohistochemical analyses suggest that proteolytic processing at the *C*‐terminal side of Arg‐36 does not occur in the pileus of *C. cylindracea*.

Recombinant Cc‐PRI3(37–95) showed activity against three kinds of fungi examined here: it inhibited mycelial growth of *A. nidulans*, *Alternaria porri*, and *F. oxysporum*, with IC_50_ values of 5–39 μm. Heat treatment of the recombinant protein in the presence of dithiothreitol indicated that the multiple disulfide bridges in recombinant Cc‐PRI3(37–95) yield a thermally stable structure that is required for the antifungal activity. The synthetic peptide composed of the residues from Leu‐21 to Arg‐36 did not show antifungal activity, nor did it affect the antifungal activity of recombinant Cc‐PRI3(37–95). Therefore, it is likely that those residues constitute an inert pro‐peptide region in native Cc‐PRI3, while Cc‐PRI3(37–95) is the mature domain with antifungal activity. Details of the proteolytic mechanism remain to be clarified.

Considering Cys‐rich antimicrobial proteins, defensins and thionins have been studied well. Defensins are cationic small proteins (< 10 kDa) produced by essentially all eukaryotes; they contain 6, 8, or 10 cysteine residues and share a similar three‐dimensional structure, despite considerable sequence variation [47, 48, 49]. Two mushroom defensins, copsin isolated from the basidiomycete *Coprinopsis cinerea* [7, 8, 10] and plectasin from the ascomycete *Pseudoplectania nigrella* [11], show antibacterial activities toward Gram‐positive bacteria: neither of them has sequence similarity to Cc‐PRI3. Thionins are small (~ 5 kDa) proteins produced by plants; they contain 6 or 8 cysteine residues and are highly homologous in their primary structures and their three‐dimensional structures [50]. The antimicrobial activities of defensins and thionins have been largely attributed to their ability to permeabilize and disrupt membranes, which initially occurs through association with bacterial or fungal cell membranes via electrostatic interactions between cationic charges on the proteins and anionic charges of microbial phospholipids [47, 48, 49, 50, 51]. In the antifungal activity of recombinant Cc‐PRI3(37–95), the cationic residues (Lys‐50, Arg‐58, Lys‐71, Arg‐72, Arg‐79, Lys‐94) that are conserved among PRI3 proteins are likely involved in targeting to the fungal plasma membrane. Treatment of the *A. nidulans* hyphae with the recombinant protein resulted in extensive PI‐staining (but not CFDA‐staining), increased leakage of intracellular nucleotides and proteins, and decreased DPH binding. These results suggest that recombinant Cc‐PRI3(37–95) disrupted cell membranes, leading to reduced viability of the hyphal cells.

The recombinant Cc‐PRI3(37–95) also appeared to cause substantial damage to the cell wall. The recombinant protein increased hyphal branching, tip swelling, and balloon formation of *A. nidulans*. The recombinant protein also inhibited conidiogenesis of *A. nidulans* and caused abnormal conidiophore morphologies. These morphological abnormalities of filamentous fungi are related to defects in cell wall integrity [52]. The cell walls of fungi are primarily composed of chitin, glucans, mannans, and glycoproteins [52, 53]. It has been reported that disruption of the gene encoding mannan synthase (CmsA or CmsB) [54], chitin‐synthase E [55], or GDP‐mannose pyrophosphorylase [56] in *Aspergillus fumigatus* results in repressed hyphal extension, abnormal hyphal branching, increased balloon formation, and aberrant conidiophore formation. Disruption of the gene encoding a chitin synthase (CsmA or CsmB) [57, 58] in *A. nidulans* has been reported to cause growth defects, frequent balloon formation along the hyphae, and aberrant conidiophore formation. In light of these observations, the results obtained here suggest that the recombinant Cc‐PRI3(37–95) might affect cell wall polysaccharide synthesis and/or the maintenance of cell wall integrity in *A. nidulans*. The more frequent balloon formation of *A. nidulans* treated with the recombinant Cc‐PRI3(37–95) on solid PDA medium than in liquid PDB medium might be explained as follows. Because solid PDA medium containing agar presented a more substantial barrier to hyphal elongation than did liquid PDB medium, damage to the fungal cell walls and cell membranes would be more severe on PDA medium, resulting in a higher frequency of balloon formation.

Some defensins target both the plasma membrane and the cell wall of fungi. For example, the Hc‐AFP defensins in *Brassicaceae* species show antifungal activity against *Botrytis cinerea* and *Fusarium solani*, causing hyper‐branching and tip swelling of the hyphae and increase in membrane permeability [59]. NaD1, a plant defensin from *Nicotiana alata*, shows antifungal activity against *F. oxysporum*, through specific interaction with a receptor in the fungal cell wall, followed by permeabilization of the plasma membrane and entry of NaD1 into the cytoplasm to cause cell death by interaction with intracellular targets [60]. Further work is required to understand the molecular mechanisms by which the recombinant Cc‐PRI3(37–95) protein exerts antifungal activity through damaging fungal plasma membranes and cell walls.

## Conclusions

Considering its antifungal activity and specific expression at the pileus surface of the immature fruiting body, the Cc‐PRI3 protein might play an important role in host defense of the fruiting body against pathogenic invaders during spore formation. The three‐dimensional molecular structure of the protein, including the disulfide connectivity, needs to be determined experimentally for understanding of the structure–activity relationship of the protein and its relationships with other cationic Cys‐rich antifungal proteins such as defensins. Finally, we propose to name the Cc‐PRI3 protein “cylindracin” after the mushroom name “*Cyclocybe cylindracea*,” for the following reasons. The gene is specifically expressed in the pileus of the fruiting body but not in the primordia of the mushroom. Meanwhile, in addition to being used for the antifungal protein agrocybin, the name “agrocybin” has been used for an antibacterial and antifungal polyacetylene compound, 8‐hydroxy‐2,4,6‐octatriynamide (PubChem CID 11004, https://pubchem.ncbi.nlm.nih.gov/compound/Agrocybin), obtained from *Agrocybe* sp. [61, 62, 63]. Another similar name, “agrocybynes,” has been used for unsaturated organic compounds that act as plant growth regulators [64, 65]. Consistent use of the name “cylindracin” for the Cc‐PRI3 protein would be helpful to avoid confusion.

## Conflict of interest

The authors declare no conflict of interest.

### Peer review

The peer review history for this article is available at https://www.webofscience.com/api/gateway/wos/peer‐review/10.1002/2211‐5463.13910.

## Author contributions

SA conceived and supervised the study; YK performed recombinant protein preparation, antibiotic analyses, and fluorescence microscopy; CA performed cDNA cloning, gene expression analysis, and immunohistochemical analysis; KE, TO, and HO contributed to antifungal assays and discussion; MS contributed to immunohistochemical analysis; MI provided technical and scientific information for cultivation of mushrooms; YK and SA wrote the manuscript.

## Supporting information


**Fig. S1.** Nucleotide sequence of *Cc*‐*Pri3* cDNA from *Cyclocybe cylindracea*. The translational start and stop codons are indicated (asterisks). Digoxigenin (DIG)‐labeled sense and antisense RNA probes for northern blotting and *in situ* hybridization were prepared using the DNA sequence underlined with the solid line. The *Cc‐Pri3* (cylindracin: alternatively proposed name in this study) sequence was deposited in the DNA Data Bank of Japan (DDBJ) with accession number LC811956.

**Fig. S2.** Preparation of recombinant Cc‐PRI3(21–95)‐Myc‐6 × His. A) Amino acid sequences of recombinant Cc‐PRI3(21–95)‐Myc‐6 × His and Cc‐PRI3(37–95)‐Myc‐6 × His. Cc‐PRI3(21–95)‐Myc‐6 × His contains the residues from Leu‐21 to Ala‐95 of the Cc‐PRI3 protein, followed by Myc‐tag and 6 × His‐tag sequences. Cc‐PRI3(37–95)‐Myc‐6 × His contains the residues from Ala‐37 to Ala‐95 of Cc‐PRI3, followed by Myc‐tag and 6 × His‐tag sequences. The DNA sequence coding for residues 21–95 of the Cc‐PRI3 protein [Cc‐PRI3(21–95)] was codon‐optimized based on codon usage in *Pichia pastoris* and was chemically synthesized, subcloned into an expression vector pPICZαA, and expressed in *P. pastoris* X‐33 cells. The Myc‐tag and 6 × His‐tag sequences are coded in the expression vector. B) SDS/PAGE analysis of recombinant proteins in the culture medium of *Pichia pastoris* X‐33 cells. Recombinant proteins in the culture medium were purified by His‐tag affinity chromatography, resolved by SDS/PAGE and detected using Coomassie Brilliant Blue staining (CBB) (*n* = 2). Proteins in the polyacrylamide gel were transferred onto a membrane and detected using an anti‐His‐tag antibody (WB) (*n* = 1). Both CBB staining and immunostaining gave two bands with apparent molecular weights of 11 and 12 kDa respectively. Amino acid sequence analysis of the proteins blotted on the membrane showed that the product giving the upper band had the expected *N*‐terminal amino acid sequence starting with Leu‐21, whereas the product giving the lower band started with Ala‐37 (i.e., it lacked the expected *N*‐terminal 16 residues). The results indicated that about half of the recombinant Cc‐PRI3(21–95)‐Myc‐6 × His protein was truncated at the *C*‐terminal side of Arg‐36 by an unknown proteolytic enzyme. Consequently, the product giving the lower molecular weight band in the SDS/PAGE, referred to as Cc‐PRI3(37–95)‐Myc‐6 × His.

**Fig. S3.** Antimicrobial assay of recombinant Cc‐PRI3(37–95) against bacteria and yeasts in liquid medium. *E. coli* DH5α or *M. luteus* was cultivated in 3% (w/v) dry bouillon, with or without recombinant Cc‐PRI3(37–95) (0.4 mg/mL), at 30°C for 24 h (*n* = 3). The yeasts *Saccharomyces cerevisiae* sigma 1278b, *Schizosaccharomyces japonicus* NRRL Y‐1026E, *P. pastoris* X‐33, and *P. pastoris* SMD1168H were cultivated in YPD medium with or without the recombinant protein (0.4 mg/mL) at 30°C for 24 h (*n* = 3). Absorbance at 620 nm was monitored every 2 h. Each experiment was performed in triplicate, and the mean values of the absorbance were plotted. Recombinant Cc‐PRI3(37–95) did not inhibit the growth of the bacteria and yeasts.

**Fig. S4.** Antimicrobial assay of recombinant Cc‐PRI3(37–95) against bacteria and yeasts on solid medium. *E. coli* DH5α, *M. luteus*, *Saccharomyces cerevisiae* sigma 1278b, *Schizosaccharomyces japonicus* NRRL Y‐1026E, or *P. pastoris* X‐33 was inoculated onto PDA medium containing 3% (w/v) dry bouillon in a 100‐mm dish. Paper disks containing 200 μg each of recombinant Cc‐PRI3(37–95) or buffer only (C: control) were placed on the solid medium. After incubation at 30°C overnight, no halo‐zone was observed around the disks (*n* = 1), indicating that recombinant Cc‐PRI3(37–95) did not inhibit the growth of the tested bacteria and yeasts.

**Video S1.** Time‐lapse images of PI staining during the hyphal growth of *A. nidulans*. The images were captured every 30 min for 24 h, in a medium containing 0.1 mg/mL Cc‐PRI3(37–95), 3% (w/v) dry bouillon, 2% glucose and 1.4 mM propidium iodide (PI) (*n* = 3).

## Data Availability

The data that supports the findings of this study are contained within the article or Supporting Information. The cylindracin (*Cc‐Pri3*) sequence was deposited in the DNA Data Bank of Japan (DDBJ) with accession number LC811956.
