# Supplementary material for: Cylindracin, a Cys‐rich protein expressed in the fruiting body of Cyclocybe cylindracea, inhibits growth of filamentous fungi but not yeasts or bacteria
Source: FEBS Open Bio. 2024 Oct 8;14(11):1805–24. doi: 10.1002/2211-5463.13910 (PMC11532979; doi:10.1002/2211-5463.13910)
Supplement: Supplementary file 1 — Fig. S1. Nucleotide sequence of Cc‐Pri3 cDNA from Cyclocybe cylindracea. The translational start and stop codons are indicated (asterisks). Digoxigenin (DIG)‐labeled sense and antisense RNA probes for northern blotting and in situ hybridization were prepared using the DNA sequence underlined with the solid line. The Cc‐Pri3 (cylindracin: alternatively proposed name in this study) sequence was deposited in the DNA Data Bank of Japan (DDBJ) with accession number LC811956. [file FEB4-14-1805-s003.pdf]

***

1 GACCGTCAACTGGATATCCCGTCTAATCCATCAAGTAGGGGCCTTCTTATTCTGCAAATCGCGATGAGGATTTCGACCGCCTTTGTAACTTTGACCTGCG 100(37)

101 TTCTCGCGACCATGGTGGTTGCTCTACCTCCTGGTCCAACGTCGCTGGAGGTCGAGGCGCTAGAAGGCCGCGCTAACGACCCGCAGTGTTTGTACGGCAA 200(137)

201 TGTCGCGGGAAAATTTTGCGACAATCAGGGCTGCAGAGATGGCGGAGGCTACTGCCAGTACAATGCACAAACTAAAAGATGCTCGATGGTGAACATGCGT 300(237)

***

301 GGAAATAGTGCACCCGTCGGCTGTCTCTCATGCACCTGCATAAAGGCGTAGGAATTAGCCACATAGGGTGCGCATAGTCGGTATAGAACTCGGATGTTGG 400(288)

401 TTTTGTTGCTCGAAGGGGTGTGCGTTTCGAAACTTCAAAAAAAAAAAAAAAAAAAAA 457

**Figure S1. Nucleotide sequence of *Cc-Pri3* cDNA from *Cyclocybe cylindracea*.**

The translational start and stop codons are indicated (asterisks). Digoxigenin (DIG)-labeled sense and antisense RNA probes for northern blotting and *in situ* hybridization were prepared using the DNA sequence underlined with the solid line. The *Cc-Pri3* (cylindracin: alternatively proposed name in this study) sequence was deposited in the DNA Data Bank of Japan (DDBJ) with accession number LC811956.
