# Supplementary material for: Cylindracin, a Cys‐rich protein expressed in the fruiting body of Cyclocybe cylindracea, inhibits growth of filamentous fungi but not yeasts or bacteria
Source: FEBS Open Bio. 2024 Oct 8;14(11):1805–24. doi: 10.1002/2211-5463.13910 (PMC11532979; doi:10.1002/2211-5463.13910)
Supplement: Supplementary file 2 — Fig. S2. Preparation of recombinant Cc‐PRI3(21–95)‐Myc‐6 × His. A) Amino acid sequences of recombinant Cc‐PRI3(21–95)‐Myc‐6 × His and Cc‐PRI3(37–95)‐Myc‐6 × His. Cc‐PRI3(21–95)‐Myc‐6 × His contains the residues from Leu‐21 to Ala‐95 of the Cc‐PRI3 protein, followed by Myc‐tag and 6 × His‐tag sequences. Cc‐PRI3(37–95)‐Myc‐6 × His contains the residues from Ala‐37 to Ala‐95 of Cc‐PRI3, followed by Myc‐tag and 6 × His‐tag sequences. The DNA sequence coding for residues 21–95 of the Cc‐PRI3 protein [Cc‐PRI3(21–95)] was codon‐optimized based on codon usage in Pichia pastoris and was chemically synthesized, subcloned into an expression vector pPICZαA, and expressed in P. pastoris X‐33 cells. The Myc‐tag and 6 × His‐tag sequences are coded in the expression vector. B) SDS/PAGE analysis of recombinant proteins in the culture medium of Pichia pastoris X‐33 cells. Recombinant proteins in the culture medium were purified by His‐tag affinity chromatography, resolved by SDS/PAGE and detected using Coomassie Brilliant Blue staining (CBB) (n = 2). Proteins in the polyacrylamide gel were transferred onto a membrane and detected using an anti‐His‐tag antibody (WB) (n = 1). Both CBB staining and immunostaining gave two bands with apparent molecular weights of 11 and 12 kDa respectively. Amino acid sequence analysis of the proteins blotted on the membrane showed that the product giving the upper band had the expected N‐terminal amino acid sequence starting with Leu‐21, whereas the product giving the lower band started with Ala‐37 (i.e., it lacked the expected N‐terminal 16 residues). The results indicated that about half of the recombinant Cc‐PRI3(21–95)‐Myc‐6 × His protein was truncated at the C‐terminal side of Arg‐36 by an unknown proteolytic enzyme. Consequently, the product giving the lower molecular weight band in the SDS/PAGE, referred to as Cc‐PRI3(37–95)‐Myc‐6 × His. [file FEB4-14-1805-s001.pdf]

A

Cc-PRI3(21-95)-Myc-6XHis: LPPGPTSLEVEALEGRANDPQCLYGNVAGKFCDNQGCRDGGGYCQYNAQTKRCSMVNMRGNSAPVGCLSCTCIKA-*Myc*-*6XHis*

Cc-PRI3(37-95)-Myc-6XHis: ANDPQCLYGNVAGKFCDNQGCRDGGGYCQYNAQTKRCSMVNMRGNSAPVGCLSCTCIKA-*Myc*-*6XHis*

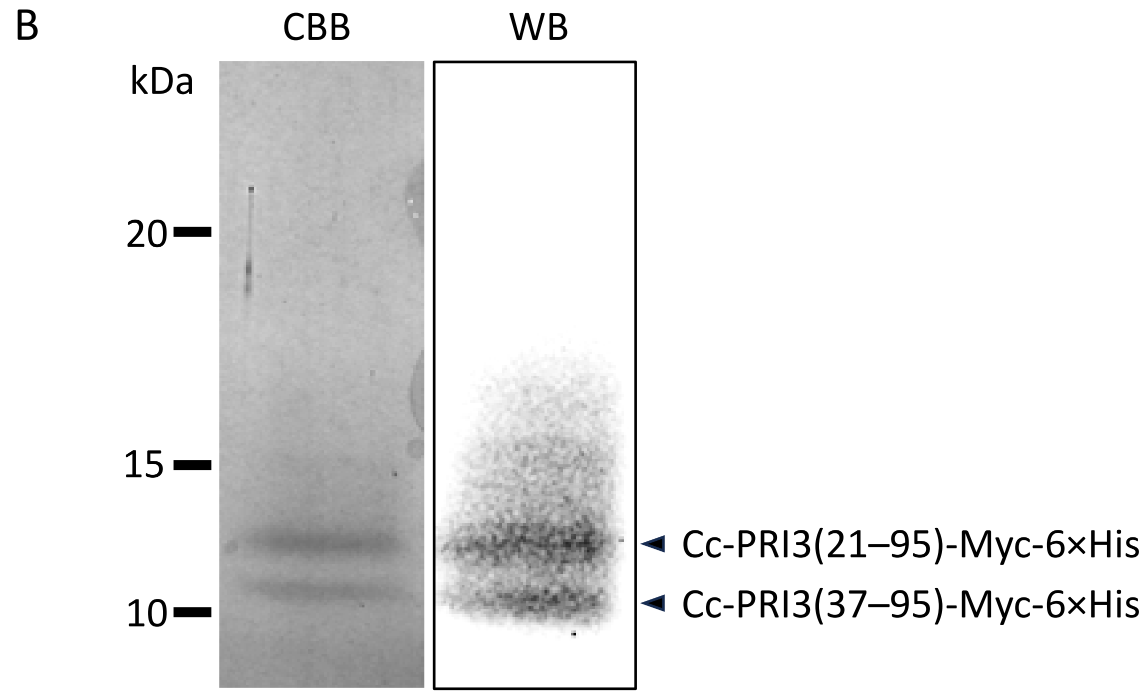

Figure S2. Preparation of recombinant Cc-PRI3(21–95)-Myc-6×His.

A) Amino acid sequences of recombinant Cc-PRI3(21–95)-Myc-6×His and Cc-PRI3(37–95)-Myc-6×His. Cc-PRI3(21–95)-Myc-6×His contains the residues from Leu-21 to Ala-95 of the Cc-PRI3 protein, followed by Myc-tag and 6×His-tag sequences. Cc-PRI3(37–95)-Myc-6×His contains the residues from Ala-37 to Ala-95 of Cc-PRI3, followed by Myc-tag and 6×His-tag sequences. The DNA sequence coding for residues 21–95 of the Cc-PRI3 protein [Cc-PRI3(21–95)] was codon-optimized based on codon usage in *Pichia pastoris* and was chemically synthesized, subcloned into an expression vector pPICZαA, and expressed in *P. pastoris* X-33 cells. The Myc-tag and 6×His-tag sequences are coded in the expression vector.

B) SDS-PAGE analysis of recombinant proteins in the culture medium of *Pichia pastoris* X-33 cells. Recombinant proteins in the culture medium were purified by His-tag affinity chromatography, resolved by SDS-PAGE and detected using Coomassie Brilliant Blue staining (CBB) ($n = 2$). Proteins in the polyacrylamide gel were transferred onto a membrane and detected using an anti-His-tag antibody (WB) ($n = 1$). Both CBB staining and immunostaining gave two bands with apparent molecular weights of 11 and 12 kDa respectively. Amino acid sequence analysis of the proteins blotted on the membrane showed that the product giving the upper band had the expected *N*-terminal amino acid sequence starting with Leu-21, whereas the product giving the lower band started with Ala-37 (i.e., it lacked the expected *N*-terminal 16 residues). The results indicated that about half of the recombinant Cc-PRI3(21–95)-Myc-6×His protein was truncated at the *C*-terminal side of Arg-36 by an unknown proteolytic enzyme. Consequently, the product giving the lower molecular weight band in the SDS-PAGE, referred to as Cc-PRI3(37–95)-Myc-6×His.
