# Supplementary material for: Cylindracin, a Cys‐rich protein expressed in the fruiting body of Cyclocybe cylindracea, inhibits growth of filamentous fungi but not yeasts or bacteria
Source: FEBS Open Bio. 2024 Oct 8;14(11):1805–24. doi: 10.1002/2211-5463.13910 (PMC11532979; doi:10.1002/2211-5463.13910)
Supplement: Supplementary file 3 — Fig. S3. Antimicrobial assay of recombinant Cc‐PRI3(37–95) against bacteria and yeasts in liquid medium. E. coli DH5α or M. luteus was cultivated in 3% (w/v) dry bouillon, with or without recombinant Cc‐PRI3(37–95) (0.4 mg/mL), at 30°C for 24 h (n = 3). The yeasts Saccharomyces cerevisiae sigma 1278b, Schizosaccharomyces japonicus NRRL Y‐1026E, P. pastoris X‐33, and P. pastoris SMD1168H were cultivated in YPD medium with or without the recombinant protein (0.4 mg/mL) at 30°C for 24 h (n = 3). Absorbance at 620 nm was monitored every 2 h. Each experiment was performed in triplicate, and the mean values of the absorbance were plotted. Recombinant Cc‐PRI3(37–95) did not inhibit the growth of the bacteria and yeasts. [file FEB4-14-1805-s005.pdf]

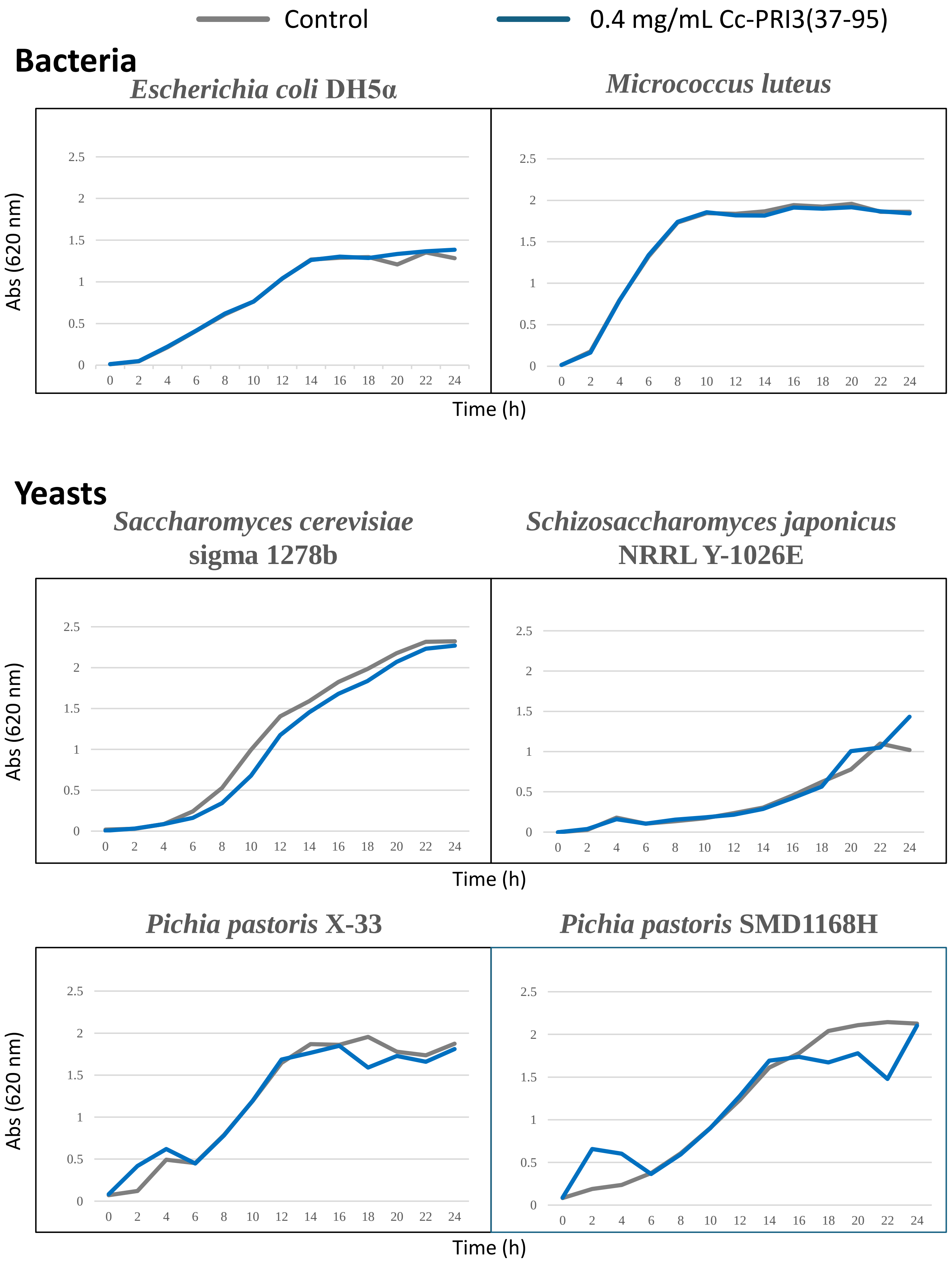

Figure S3. Antimicrobial assay of recombinant Cc-PRI3(37–95) against bacteria and yeasts in liquid medium. *E. coli* DH5α or *M. luteus* was cultivated in 3% (w/v) dry bouillon, with or without recombinant Cc-PRI3(37–95) (0.4 mg/ml), at 30°C for 24 h (n = 3). The yeasts *Saccharomyces cerevisiae* sigma 1278b, *Schizosaccharomyces japonicus* NRRL Y-1026E, *P. pastoris* X-33, and *P. pastoris* SMD1168H were cultivated in YPD medium with or without the recombinant protein (0.4 mg/ml) at 30°C for 24 h (n = 3). Absorbance at 620 nm was monitored every 2 hours. Each experiment was performed in triplicate, and the mean values of the absorbance were plotted. Recombinant Cc-PRI3(37–95) did not inhibit the growth of the bacteria and yeasts.
