# Supplementary material for: Cylindracin, a Cys‐rich protein expressed in the fruiting body of Cyclocybe cylindracea, inhibits growth of filamentous fungi but not yeasts or bacteria
Source: FEBS Open Bio. 2024 Oct 8;14(11):1805–24. doi: 10.1002/2211-5463.13910 (PMC11532979; doi:10.1002/2211-5463.13910)
Supplement: Supplementary file 4 — Fig. S4. Antimicrobial assay of recombinant Cc‐PRI3(37–95) against bacteria and yeasts on solid medium. E. coli DH5α, M. luteus, Saccharomyces cerevisiae sigma 1278b, Schizosaccharomyces japonicus NRRL Y‐1026E, or P. pastoris X‐33 was inoculated onto PDA medium containing 3% (w/v) dry bouillon in a 100‐mm dish. Paper disks containing 200 μg each of recombinant Cc‐PRI3(37–95) or buffer only (C: control) were placed on the solid medium. After incubation at 30°C overnight, no halo‐zone was observed around the disks (n = 1), indicating that recombinant Cc‐PRI3(37–95) did not inhibit the growth of the tested bacteria and yeasts. [file FEB4-14-1805-s002.pdf]

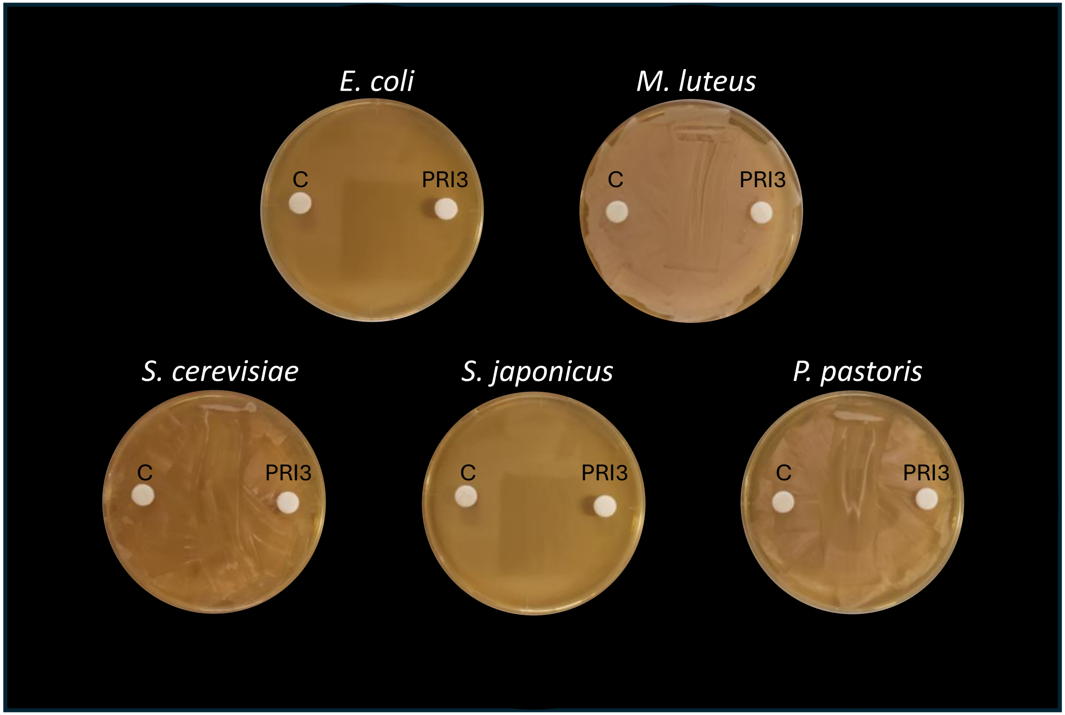

Figure S4. Antimicrobial assay of recombinant Cc-PRI3(37–95) against bacteria and yeasts on solid medium. *E. coli* DH5α, *M. luteus*, *Saccharomyces cerevisiae* sigma 1278b, *Schizosaccharomyces japonicus* NRRL Y-1026E, or *P. pastoris* X-33 was inoculated onto PDA medium containing 3% (w/v) dry bouillon in a 100-mm dish. Paper disks containing 200 μg each of recombinant Cc-PRI3(37–95) or buffer only (C: control) were placed on the solid medium. After incubation at 30°C overnight, no holo-zone was observed around the disks (n = 1), indicating that recombinant Cc-PRI3(37–95) did not inhibit the growth of the tested bacteria and yeasts.
